# Temporal, Phenotypic, and Treatment-Context Heterogeneity in Retinal Vasculitis Reporting with Pegcetacoplan: A United States Pharmacovigilance Study

**DOI:** 10.3390/jpm16100533

**Published:** 2026-10-16

**Authors:** Siddharth Gandhi, Michael Balas, Patrick Wang, Parnian Arjmand

**Affiliations:** 1 School of Medicine, Queen’s University, Kingston, ON K7L 3L4, Canada; 2 Department of Ophthalmology and Vision Sciences, University of Toronto, Toronto, ON M5T 3A9, Canada; 3 Department of Ophthalmology, Queen’s University, Kingston, ON K7L 3L4, Canada; 4 Mississauga Retina Institute, Mississauga, ON M5T 3A9, Canada

**Keywords:** geographic atrophy, age-related macular degeneration, pegcetacoplan, retinal vasculitis, pharmacovigilance, medication safety, personalized medicine, anti-VEGF, FAERS

## Abstract

**Background/Objectives:** Pegcetacoplan slows geographic atrophy (GA) progression, but postmarketing retinal vasculitis remains an important safety concern. We evaluated whether retinal vasculitis reporting changed over time, whether the reported phenotype changed with respect to occlusive coding, and whether vasculitis was associated with identifiable treatment contexts. **Methods:** We analyzed the United States (US) Food and Drug Administration Adverse Event Reporting System (FAERS) data from 17 February 2023 through 31 December 2025. Strict retinal vasculitis reports required pegcetacoplan to be coded as the primary-suspect drug. Faricimab was used as a descriptive contemporaneous intravitreal comparator because US exposure estimates could be constructed over the same period; it was not treated as a safety-neutral control. Separately, within all US primary-suspect Syfovre reports, we examined whether another anti-vascular endothelial growth factor (anti-VEGF) agent was co-reported. These anti-VEGF agents were evaluated as treatment-context variables rather than as temporal comparators. **Results:** We identified 79 US primary-suspect Syfovre retinal vasculitis reports, of which 58 had quarter-assignable clinical event dates. Overall, 42/79 (53.2%) contained an occlusive-specific vasculitis preferred term and 37/79 (46.8%) did not. Pegcetacoplan reporting declined from 4.09 to 0.38 reports per 10,000 estimated vials between the pre-change and post-change periods (reporting-rate ratio 0.09, 95% confidence interval [CI] 0.05–0.16; *p* < 0.001). Faricimab reporting also declined; the ratio of reporting-rate ratios was 0.79 (95% CI 0.22–2.78; *p* = 0.711), with wide uncertainty. Among temporally assignable pegcetacoplan reports, occlusive-specific coding decreased from 16/21 (76.2%) before the change to 8/30 (26.7%) afterward (*p* < 0.001). Another anti-VEGF agent was listed in 19/79 vasculitis reports versus 50/571 nonvasculitis reports (reporting odds ratio 3.30, 95% CI 1.83–5.96; *p* < 0.001); however, adjustment for medication count and report completeness attenuated the estimate to 1.89 (95% CI 0.99–3.61; *p* = 0.052). **Conclusions:** Pegcetacoplan retinal vasculitis reporting declined over time, but the descriptive faricimab comparison was too imprecise to determine whether the proportional temporal change differed between products. The reported phenotype shifted away from occlusive-specific coding, while the anti-VEGF co-reporting association was sensitive to adjustment for documentation intensity. These findings characterize heterogeneity in spontaneous safety reporting and do not establish patient-level risk, changes in clinical event severity, or causal treatment effects.

## 1. Introduction

Geographic atrophy (GA) secondary to age-related macular degeneration is a progressive cause of irreversible central visual loss. Pegcetacoplan (Syfovre), an inhibitor of complement component C3, was approved by the United States (US) Food and Drug Administration (FDA) on 17 February 2023 for intravitreal treatment of GA [1]. In the phase 3 OAKS and DERBY trials, monthly and every-other-month dosing reduced GA lesion growth compared with sham at 24 months, although key secondary visual function outcomes did not differ between groups [2]. Subsequent imaging analyses have demonstrated heterogeneity in disease progression and treatment response according to lesion topography and retinal structural features [3,4]. These findings support an individualized treatment decision that weighs expected anatomic benefit, treatment burden, visual status, and safety [2,5].

Postmarketing safety has also become an important consideration in pegcetacoplan treatment. Soon after commercial introduction, retinal vasculitis was reported following intravitreal pegcetacoplan. The American Society of Retina Specialists (ASRS) Research and Safety in Therapeutics (ReST) Committee described 14 affected eyes in 13 patients, including 11 eyes with occlusive retinal vasculopathy and substantial visual morbidity in some cases [6]. FDA subsequently updated Syfovre labeling in November 2023 to include retinal vasculitis and retinal vascular occlusion in the warnings and postmarketing sections [7]. During its investigation, the manufacturer identified structural variation in a 19-gauge filter needle supplied in certain injection kits and, on 22 August 2023, recommended discontinuing kits containing that needle in favor of an 18-gauge filter needle. The manufacturer stated that a causal relationship between the filter-needle variation and retinal vasculitis had not been established [8].

Spontaneous-reporting data make it difficult to interpret safety signals around product or delivery-system changes. Reporting can vary with product age, regulatory attention, and publicity, and these effects are not consistent across products [9,10]. A simple before-and-after comparison may therefore suggest a product-specific change when reporting behavior is also changing over time. Aggregate report counts may also obscure changes in the type of events being reported or the treatment context in which they occur.

Prior studies using the FDA Adverse Event Reporting System (FAERS) have already characterized ocular-adverse-event reporting with complement inhibitors, including pegcetacoplan [11], and retinal vasculitis reporting across multiple intravitreal agents, including pegcetacoplan and faricimab [12]. The present study was therefore not designed to identify the pegcetacoplan retinal vasculitis signal de novo. Its incremental contribution is to examine three additional dimensions of that established signal: temporal changes in reporting around the August 2023 filter-needle recommendation, changes in occlusion-associated coding among reported vasculitis cases, and anti-VEGF co-reporting within the broader Syfovre safety-report population.

Treatment context is particularly relevant in GA because some pegcetacoplan-treated patients have pre-existing or incident neovascular age-related macular degeneration (nAMD) requiring anti-vascular endothelial growth factor (anti-VEGF) therapy. New-onset exudation was observed during clinical development, and real-world series have documented pegcetacoplan use in eyes with prior or concurrent anti-VEGF management [13,14,15].

Examining whether postmarketing reporting varies by time, event phenotype, or treatment context may help characterize the safety signal and identify priorities for prospective study, even though spontaneous reports cannot quantify individual risk.

We performed an expanded pharmacovigilance study with three objectives. First, we quantified temporal changes in retinal vasculitis reporting after the August 2023 filter-needle recommendation and compared them descriptively with contemporaneous reporting for the intravitreal agent faricimab. Second, we assessed whether the reported vasculitis phenotype shifted with respect to occlusive coding. Third, we constructed a US Syfovre case–noncase cohort to examine whether retinal vasculitis was disproportionately represented in identifiable treatment contexts, with particular attention to co-reporting of another anti-VEGF agent. These analyses extend prior FAERS studies by focusing specifically on temporal reporting patterns, occlusion-associated coding, and anti-VEGF treatment-context co-reporting rather than on detection of the vasculitis signal itself. The temporal analysis was considered primary; the phenotype and case–noncase analyses were interpreted as secondary and hypothesis-generating.

## 2. Materials and Methods

### 2.1. Study Design and Data Sources

We conducted a retrospective pharmacovigilance study using publicly available FAERS quarterly data from 17 February 2023, the date of the US approval of Syfovre, through 31 December 2025. Quarterly American Standard Code for Information Interchange (ASCII) extracts were linked across demographic and administrative (DEMO), drug (DRUG), reaction (REAC), outcome (OUTC), report-source (RPSR), therapy (THER), and indication (INDI) tables [16]. Because FAERS is a spontaneous-reporting system rather than a population-based clinical registry, all analyses were designed to characterize reporting patterns rather than clinical incidence or causal drug effects. The study used publicly available, de-identified data and did not require institutional ethics review.

### 2.2. Case Identification, Relational Enrichment, and Deduplication

To maximize sensitivity during case retrieval, candidate pegcetacoplan and faricimab case identifiers (CASEIDs) were assembled from drug-specific searches containing retinal vasculitis or retinal vascular-occlusion reaction terms. Every candidate CASEID was then re-linked to the FAERS relational tables and reclassified using a strict final case definition. A report qualified as retinal vasculitis only if the latest report version contained at least one of the following Medical Dictionary for Regulatory Activities (MedDRA) preferred terms: retinal vasculitis, retinal occlusive vasculitis, haemorrhagic occlusive retinal vasculitis, or hemorrhagic occlusive retinal vasculitis. Retinal artery, retinal vein, or retinal vascular occlusion without a retinal vasculitis preferred term was not sufficient to define a vasculitis case because vascular occlusion alone does not necessarily indicate an inflammatory vasculitis. However, when one of these vascular-occlusion terms occurred within a report that already met the strict retinal vasculitis definition, it was incorporated into the expanded occlusion-associated phenotype sensitivity analysis described below.

FAERS follow-up submissions can create multiple versions of the same report [16]. We identified the latest version for each FAERS case identifier using the report-version number, FDA receipt date, and report identifier, then retained the relational records linked to that latest version. Because separate FAERS case identifiers can still represent duplicate submissions of the same clinical event, we also screened for potential cross-identifier duplication within each drug cohort. Report pairs were flagged if they shared the same manufacturer report number or if they matched on sex, age, clinical event date, and reporter country and had at least 50% overlap in reported reaction terms. No potential cross-identifier duplicate pairs were identified by these criteria. Because identifying information in FAERS is incomplete, however, unrecognized duplication across different case identifiers cannot be excluded. Accordingly, the unit of analysis throughout this study is a deduplicated FAERS report, not a verified unique patient or eye. The duplicate and cohort-overlap audit is provided in Supplementary Table S1.

The primary temporal analysis required the target drug to be coded as the primary suspect. A sensitivity analysis allowed either primary- or secondary-suspect target-drug status.

For pegcetacoplan, reports explicitly naming Syfovre were accepted. Reports using the generic name pegcetacoplan were accepted only when structured route or dose text supported ocular administration, which excluded systemic pegcetacoplan products. Faricimab reports were identified through Vabysmo or faricimab naming. Because the faricimab exposure denominator was estimated from US sales, denominator-based comparator analyses were restricted to reports with reporter country coded as the United States. All strict pegcetacoplan reports contributing to the temporal cohort were US-reporter reports.

### 2.3. Exposure Denominators and Temporal Periods

Pegcetacoplan exposure was reconstructed quarterly from manufacturer-reported commercial and sample or free-goods vial distributions [17]. For Q1 2023, the manufacturer reported more than 6000 commercial vials and more than 3400 sample vials distributed by 31 March 2023; we therefore used 9400 vials as a conservative lower-bound exposure proxy for that quarter [17]. For subsequent quarters, the manufacturer-reported total distribution or the reported commercial and sample or free-goods components were used as available. Faricimab US vial volume was estimated from quarterly US product sales reported by Roche [18]. Sales reported in Swiss francs were converted to US dollars using the corresponding annual average exchange rate and divided by the US launch wholesale acquisition cost of USD 2190 per treatment, treating one treatment as one vial [19]. These quantities are exposure proxies rather than verified administered injections or treated patients and do not distinguish treatment initiation from repeat injections. Quarter-specific numbers of patients initiating either treatment were not available, so reporting rates per first injection could not be estimated. The faricimab calculation also assumes that a fixed launch price is a reasonable divisor for sales over time despite discounts, rebates, and subsequent price changes. Accordingly, denominator-based outcomes are described as estimated reporting rates. Quarter-specific event counts, exposure denominators, reporting rates, and denominator reconstruction details are provided in Supplementary Table S2. Faricimab was used only as a descriptive contemporaneous reporting comparator and was not assumed to share the same mechanism of retinal inflammation as pegcetacoplan. In November 2023, near the beginning of the defined post-change period, Genentech issued an Important Drug Warning concerning retinal vasculitis and/or retinal vascular occlusion following postmarketing reports with faricimab [20]. This communication could itself have influenced faricimab reporting during the study period.

Reports were assigned to calendar quarter using the clinical event date recorded in the drug-specific case-extraction files created during the original case review. Within each extraction file, reports were ordered by report date, and the latest available row for each case identifier was retained. Thus, the clinical event date used for the primary temporal analysis came from the earlier case-review extraction rather than being re-derived from the latest linked FAERS report version. The event date recorded in the latest linked FAERS demographic record was retained for audit purposes but was not substituted when the original temporal event date was unavailable. Reports without at least the year and month of event dating could not be assigned to a quarter and were excluded from the primary temporal analysis. Partial dates containing year and month were assigned to the corresponding quarter. Q1 through Q4 denote the first through fourth calendar quarters, respectively. The pre-change period comprised Q1 and Q2 2023. Q3 2023 was treated as a transition quarter because the 18-gauge filter-needle recommendation occurred on 22 August 2023 [8]. The post-change period comprised Q4 2023 through Q4 2025. Q3 2023 was retained in longitudinal figures but excluded from formal pre-change versus post-change comparisons. The individual filter needle used in any FAERS case was not available, and the timing of the depletion of the older injection-kit inventory was unknown. Calendar period therefore served only as an ecological proxy for the injection-kit environment. Throughout the manuscript, the terms pre-change and post-change refer to calendar periods before and after the August 2023 filter-needle recommendation and do not indicate verified patient-level exposure to a particular filter needle.

### 2.4. Occlusive- and Occlusion-Associated Vasculitis Phenotypes

Among strict primary-suspect retinal vasculitis reports, the primary occlusive-coded phenotype was assigned when any associated MedDRA preferred term was retinal occlusive-vasculitis, haemorrhagic occlusive retinal vasculitis, or hemorrhagic occlusive retinal vasculitis. Reports meeting the strict retinal vasculitis case definition without one of these preferred terms were classified as lacking occlusive-specific coding. Because clinically occlusive events may also be represented by separate vascular-occlusion terminology, we performed an expanded sensitivity analysis in which a strict retinal vasculitis report was additionally classified as occlusion-associated when retinal artery occlusion, retinal vein occlusion, or retinal vascular occlusion was also reported. These vascular-occlusion terms alone were not sufficient to qualify a report as retinal vasculitis. Both classifications therefore remained restricted to reports meeting the strict retinal vasculitis case definition. This expanded definition was intended to capture reports in which an occlusive vasculitis phenotype may have been represented by a retinal vasculitis term together with a separate vascular-occlusion term rather than by a single occlusive-vasculitis preferred term. These phenotypes reflect spontaneous-report terminology rather than independently adjudicated angiographic findings. The public FAERS ASCII files do not encode a case-level MedDRA version; preferred-term strings were therefore analyzed as supplied in the REAC table [16]. Strict and expanded phenotype analyses, exact preferred-term combinations, and preferred-term mapping are provided in Supplementary Table S3. Because terminology and coding practices may change as awareness of an adverse event evolves, differences between calendar periods may reflect changes in classification or documentation in addition to changes in the underlying clinical phenotype. We compared the pre-change and post-change proportions within each drug and evaluated drug-by-period interactions under both definitions. Q3 2023 was excluded from these comparisons.

### 2.5. US Syfovre Case–Noncase Cohort

To examine treatment-context heterogeneity without relying on vial-based exposure denominators, we separately identified all US FAERS reports during the study period in which Syfovre or ocular pegcetacoplan was coded as the primary suspect. Cases met the strict retinal vasculitis definition; all other qualifying Syfovre reports served as noncases. This analysis used the initial FDA receipt date to define the post-approval study window and therefore did not require a complete clinical event date. Consequently, its vasculitis case count was larger than the number assignable to temporal quarters.

The principal treatment-context variable was co-reporting of another anti-VEGF agent. A report was classified as anti-VEGF co-reported when faricimab, aflibercept, ranibizumab, bevacizumab, or brolucizumab appeared as a non-pegcetacoplan drug in the same FAERS report, regardless of drug-role coding. These agents were examined as markers of retinal treatment context within Syfovre reports, not as mechanistic comparators for pegcetacoplan-associated inflammation. This definition identifies documentation of another anti-VEGF agent but does not establish active co-administration, treatment chronology, same-eye exposure, or causal relevance. We additionally summarized anti-VEGF agents individually and by reported drug role. A sensitivity analysis restricted anti-VEGF exposure to agents coded as suspect or concomitant. Where structured therapy-start dates and clinical event dates were both available, their temporal sequence was summarized descriptively. These dates were not assumed to establish active treatment at the time of vasculitis, same-eye administration, or cumulative injection exposure. Agent-specific anti-VEGF drug-role and structured treatment chronology summaries are provided in Supplementary Table S4.

Additional variables included reporting era based on whether the initial FDA receipt date occurred on or after 22 August 2023, age, sex, and structured bilateral-treatment documentation derived from ocular dose text. Reporting-bias sensitivity variables included expedited-report status, direct-report status, and the presence of any coded serious outcome in OUTC. An exploratory indication variable identified structured nAMD or choroidal neovascularization terms in INDI. To characterize documentation intensity, we counted the number of unique listed drugs and reaction preferred terms per report and calculated a structured-data completeness score from the presence of nine fields: age, sex, event date, indication, outcome, route, dose, therapy-start date, and report source.

### 2.6. Statistical Analysis

**Figure 1:**
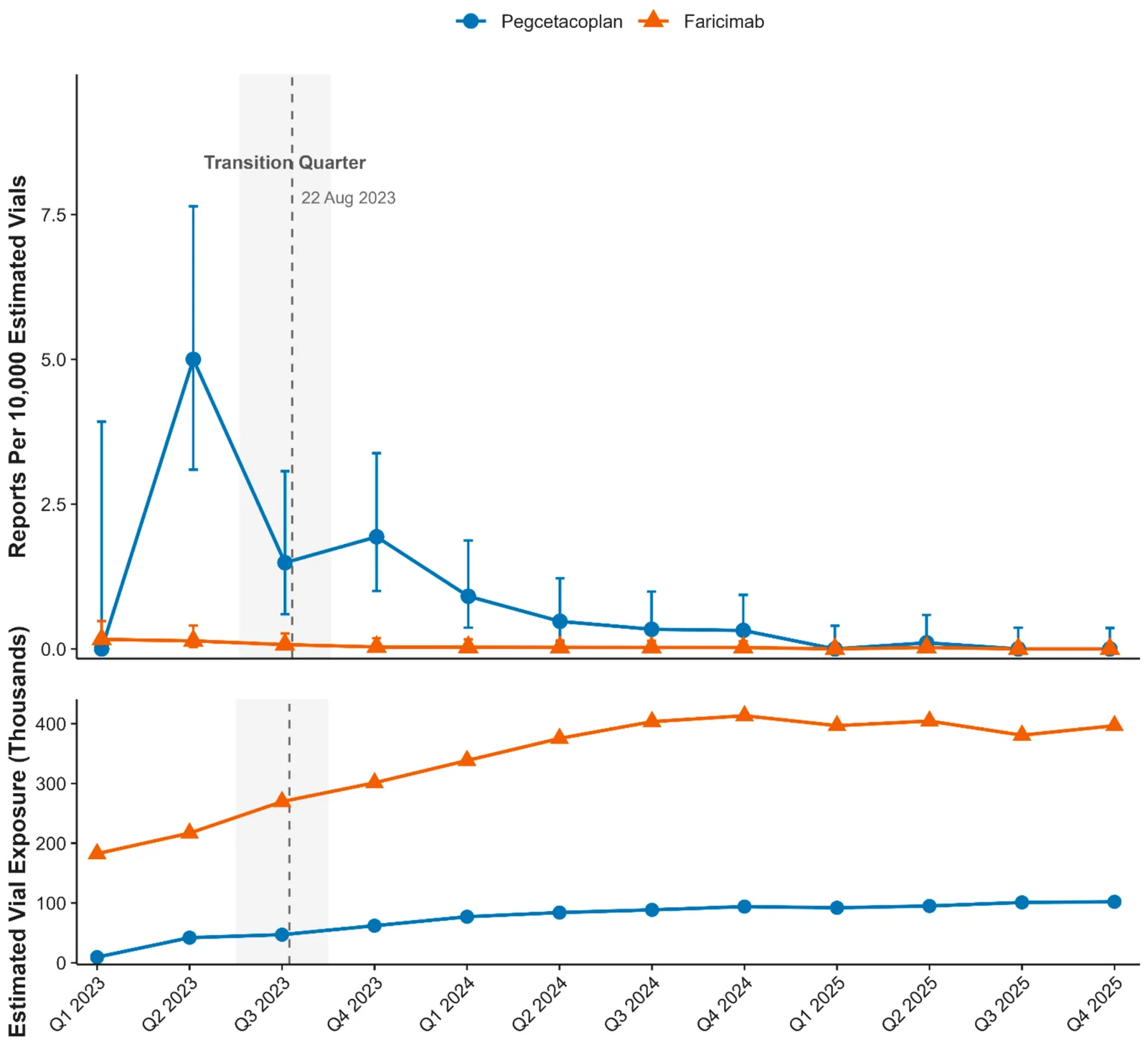
Quarterly retinal vasculitis reporting rates and estimated vial exposure for pegcetacoplan and faricimab. The upper panel shows strict primary-suspect retinal vasculitis reporting rates per 10,000 estimated vials for pegcetacoplan and the US-reporter faricimab comparator, with exact Poisson 95% confidence intervals. The lower panel shows estimated quarterly vial exposure for both agents. The shaded Q3 2023 interval represents the transition quarter, and the dashed vertical line indicates 22 August 2023, when the manufacturer recommended discontinuation of injection kits containing the 19-gauge filter needle in favor of an 18-gauge filter needle. Q3 2023 is displayed for temporal context but was excluded from formal pre-change versus post-change comparisons. Exposure quantities are reconstructed proxies rather than verified administered injections, and reporting rates should not be interpreted as clinical incidence. Abbreviations: Q1, first quarter; Q2, second quarter; Q3, third quarter; Q4, fourth quarter.

Quarterly reporting-rates were expressed per 10,000 estimated vials. Exact Poisson 95% CIs were calculated for the quarterly reporting-rates displayed in Figure 1. For each drug, pooled pre-change and post-change reporting-rate ratios (RRs) and 95% confidence intervals (CIs) were calculated on the log scale using Poisson count variance. As a descriptive contemporaneous comparison, the pegcetacoplan reporting RR was divided by the corresponding faricimab reporting RR to obtain a ratio of reporting rate ratios (RRRs), with a Wald confidence interval based on the four event counts. Faricimab was used to provide temporal context rather than as a control group or as a formal adjustment for background reporting behavior. With only two pre-change quarters, comparable underlying pre-change trends between the products could not be established. Because both exposure denominators are proxies, deterministic stress tests were performed to assess sensitivity to time-varying denominator error. The faricimab post-change denominator was varied from 70% to 130% of the reconstructed value, and the pegcetacoplan post-change denominator from 80% to 120%, while the corresponding pre-change denominator was held fixed. Uniform scaling of the faricimab denominator across all quarters was also examined and, as expected, did not change the within-drug reporting rate ratio because a constant multiplicative factor cancels from the pre-change versus post-change comparison. The primary concern was therefore time-varying rather than constant denominator error.

Confidence intervals for denominator-based reporting rates and rate ratios condition on the reconstructed exposure proxies as fixed values and therefore do not propagate uncertainty in the denominator estimates.

Temporal sensitivity analyses (1) allowed primary- or secondary-suspect target-drug status, (2) excluded the low-volume Q1 2023 launch quarter, and (3) treated Q4 2023 as part of an extended transition interval so that the post-change period began in Q1 2024. To examine the quarterly trajectory without collapsing observations into pooled pre-change and post-change periods, we also fitted exploratory Poisson regression models to quarterly event counts using the log of estimated vial exposure as an offset. Q3 2023 was excluded as the transition quarter. A level-change model included drug, calendar quarter, post-recommendation period, and a drug-by-post-period interaction. A segmented model additionally included time since the transition quarter and its interaction with the drug to assess differential post-recommendation slopes. Heteroskedasticity-consistent (HC0) standard errors were used. Because only two pre-change quarters were available and faricimab event counts were sparse, these models were considered exploratory. Full denominator stress-test and exploratory quarterly Poisson model results are provided in Supplementary Table S5.

To assess potential selection related to event-date availability, strict US primary-suspect retinal vasculitis reports with and without a quarter-assignable clinical event date were compared within each drug by initial FDA receipt period, reporter occupation, FAERS report type, coded serious outcome, strict occlusive-vasculitis coding, and report-version number. Reporter occupation, report type, and receipt-period distributions were summarized descriptively. Serious-outcome and occlusive-coding proportions were compared using two-sided Fisher exact tests, and report-version distributions were compared using the Mann–Whitney U test. Detailed event-date completeness and dated-versus-undated report characteristics are provided in Supplementary Table S6.

As a complementary temporal analysis, reports were indexed by initial FDA receipt quarter and the exposure-adjusted pre-change versus post-change analysis was repeated using the same calendar-period definitions. Because receipt date reflects when a report entered FAERS rather than when the clinical event occurred, this analysis was interpreted separately from the primary event-date analysis. Reporting lag was summarized from event quarter to initial FDA receipt quarter and, among reports with an exact clinical event date, in days from the event to initial FDA receipt. To assess incomplete ascertainment near the study cutoff, the primary event-date analysis was also repeated after excluding the final 6 months and final 12 months of observation. Receipt-date sensitivity, reporting-lag, and end-of-window analyses are provided in Supplementary Table S7.

Occlusive-coded and expanded occlusion-associated proportions were compared using two-sided Fisher exact tests. Pre-change versus post-change odds ratios (ORs) were accompanied by approximate Wald 95% CIs, and Wilson 95% CIs were calculated for descriptive proportions. Differential phenotypic change between drugs was evaluated using Firth penalized logistic regression containing drug, period, and drug-by-period interaction terms because the faricimab comparator cells were sparse. The interaction analyses were considered exploratory.

Within the Syfovre case–noncase cohort, unadjusted reporting odds ratios (RORs) were calculated from two-by-two tables with two-sided Fisher exact *p* values. The principal logistic model included anti-VEGF co-reporting and reporting era. The reporter-type model retained both variables and added expedited-report status, while the seriousness-adjusted model retained both variables and added coded serious-outcome status. Analyses restricted to serious-outcome reports, expedited reports, or expedited/direct reports each retained anti-VEGF co-reporting and reporting era as covariates. The exploratory indication-adjusted model included anti-VEGF co-reporting, reporting era, expedited-report status, and documented neovascular age-related macular degeneration or choroidal neovascularization. Additional sensitivity analyses restricted anti-VEGF exposure to agents coded as suspect or concomitant, excluded faricimab and brolucizumab from the exposure definition, restricted noncases to reports containing ocular adverse events, and restricted the analysis to reports with retinal or age-related macular degeneration indication context.

Because vasculitis reports contained more documented medications and reactions than nonvasculitis reports, an additional model included anti-VEGF co-reporting, reporting era, total number of listed drugs, and overall report completeness. A complete-case demographic sensitivity model included anti-VEGF co-reporting, reporting era, age per 10 years, and female sex. Full treatment-context sensitivity and report-completeness analyses are provided in Supplementary Table S8. Models showing sparse-data separation were suppressed rather than reporting unstable maximum-likelihood estimates. All tests were two-sided, with *p* < 0.05 considered statistically significant. No multiplicity adjustment was applied to secondary or exploratory analyses, which were interpreted as hypothesis-generating. Analyses were performed in Python 3.12.4 using pandas 3.0.5 and NumPy 2.5.1. Maximum-likelihood logistic regression, Poisson models, and two-sided Fisher exact tests were implemented in the analysis code. Firth penalized logistic regression was implemented using NumPy, with SciPy used for penalized likelihood-ratio *p* values. Figures were generated in R using ggplot2.

## 3. Results

### 3.1. Cohort Assembly and Geographic Matching

After strict MedDRA reclassification and restriction to US primary-suspect target-drug reports within the study window, 79 pegcetacoplan and 45 faricimab retinal vasculitis reports were identified. Of the 79 pegcetacoplan reports, 58 (73.4%) had quarter-assignable event dates and contributed to the temporal analysis; the remaining 21 (26.6%) lacked a quarter-assignable event date in the temporal event-date source. Of the 45 US faricimab reports, 14 (31.1%) had quarter-assignable event dates and contributed to the denominator-matched temporal comparator analysis; 31 (68.9%) did not. Among temporally assignable reports, pegcetacoplan contributed 21 pre-change reports, seven transition-quarter reports, and 30 post-change reports. The US faricimab comparator contributed six, two, and six reports, respectively (Table 1). The 79 pegcetacoplan and 45 faricimab reports had no shared FAERS case identifiers, and the cross-identifier duplicate screen identified no candidate duplicate pairs within either drug cohort. The drug-specific event counts were therefore nonoverlapping at the FAERS case-identifier level. All counts in this study refer to reports rather than verified unique patients or eyes.

Event-date completeness varied by initial FDA receipt period. For pegcetacoplan, event dates were quarter-assignable for 6/6 (100%) reports received in the pre-change period, 11/13 (84.6%) in the transition period, and 41/60 (68.3%) in the post-change period. Corresponding faricimab proportions were 1/2 (50.0%), 4/7 (57.1%), and 9/36 (25.0%). Coded serious outcomes did not differ significantly between reports with and without quarter-assignable event dates for pegcetacoplan (57/58 [98.3%] vs. 19/21 [90.5%], *p* = 0.171) or faricimab (12/14 [85.7%] vs. 24/31 [77.4%], *p* = 0.698). Strict occlusive-vasculitis coding also did not differ significantly for pegcetacoplan (28/58 [48.3%] vs. 14/21 [66.7%], *p* = 0.203) or faricimab (9/14 [64.3%] vs. 15/31 [48.4%], *p* = 0.356). Pegcetacoplan reports with quarter-assignable event dates had higher report-version numbers than those without assignable dates (median 4, interquartile range [IQR] 2–5 vs. median 1, IQR 1–3; *p* = 0.013), whereas faricimab report-version distributions were similar (median 2, IQR 1.25–3 vs. median 2, IQR 1–3; *p* = 0.503). Reporter-occupation and report-type distributions are provided in Supplementary Table S6. These comparisons characterize measured differences in event-date availability but cannot exclude selection related to unmeasured report characteristics.

The separate US Syfovre case–noncase cohort contained 650 primary-suspect reports, of which 79 met the strict retinal vasculitis definition and 571 were noncases. Forty-two of the 79 vasculitis reports (53.2%) contained an occlusive-vasculitis preferred term.

### 3.2. Temporal Changes in Reporting Rates

Pegcetacoplan retinal vasculitis reporting declined from 4.09 reports per 10,000 estimated vials during the pre-change period to 0.38 per 10,000 estimated vials after the transition quarter, corresponding to a reporting RR of 0.09 (95% CI 0.05–0.16; *p* < 0.001) (Figure 1). US-reporter faricimab reporting declined over the same calendar periods from 0.15 to 0.02 reports per 10,000 estimated vials (reporting RR 0.12, 95% CI 0.04–0.36; *p* < 0.001). The RRR was 0.79 (95% CI 0.22–2.78; *p* = 0.711). The wide confidence interval was compatible with substantial relative differences in either direction, so this descriptive comparison does not establish that pegcetacoplan and faricimab experienced similar proportional changes. Because the exposure denominators represent total vial volume, these comparisons do not account for changes over time in the proportion of treatment initiations versus repeat injections.

The original event-date sensitivity analyses were directionally consistent with the primary comparison. Allowing primary- or secondary-suspect target-drug status yielded an RRR of 0.59 (95% CI 0.18–1.96; *p* = 0.389). Excluding Q1 2023 yielded an RRR of 0.59 (95% CI 0.13–2.64; *p* = 0.493), and beginning the post-change period in Q1 2024 yielded an RRR of 0.56 (95% CI 0.15–2.15; *p* = 0.399) (Table 2). Exploratory quarterly Poisson models provided additional context for the longitudinal pattern. In the level-change model, the pegcetacoplan-by-post-period interaction was 0.73 (95% CI 0.28–1.86; *p* = 0.507), providing no clear evidence of a differential immediate level change relative to faricimab after accounting for calendar time. In the segmented model, the pegcetacoplan-by-post-period interaction was 2.90 (95% CI 1.15–7.32; *p* = 0.025), while the pegcetacoplan-specific post-period slope interaction was 0.71 per quarter (95% CI 0.56–0.89; *p* = 0.004). These estimates suggest a steeper subsequent decline in pegcetacoplan reporting relative to faricimab within the segmented specification, but they were considered exploratory because only two pre-change quarters and sparse comparator events were available.

Results were also sensitive to plausible time-varying error in the exposure proxies. Across the prespecified denominator stress tests, the RRR ranged from 0.55 to 1.02, and all corresponding 95% confidence intervals included 1. These analyses reinforce that the descriptive comparator estimate is sensitive to uncertainty in how exposure changed over time.

All 79 pegcetacoplan and 45 faricimab reports had an initial FDA receipt date and could therefore be assigned to a receipt quarter, although Q3 2023 was again excluded from the formal pre-change versus post-change comparison. When indexed by initial receipt date, the pegcetacoplan reporting RR was 0.65 (95% CI 0.28–1.50; *p* = 0.308), whereas the faricimab reporting RR was 2.11 (95% CI 0.51–8.76; *p* = 0.305), yielding an RRR of 0.31 (95% CI 0.06–1.60; *p* = 0.161). Thus, receipt-date indexing produced a different descriptive temporal pattern from event-date indexing, with substantial imprecision in the comparator-adjusted estimate.

Reporting lag was variable. Median lag from the event quarter to the initial FDA receipt quarter was 1 quarter for pegcetacoplan (IQR 0–3) and 1 quarter for faricimab (IQR 1–2.75). Among reports with exact clinical event dates, median event-to-receipt lag was 79 days (IQR 27–228; *n* = 56) for pegcetacoplan and 72 days (IQR 52–225; *n* = 13) for faricimab. Excluding the final 6 months yielded a comparator-adjusted RRR of 0.82 (95% CI 0.23–2.88; *p* = 0.753), and excluding the final 12 months yielded an RRR of 0.96 (95% CI 0.26–3.58; *p* = 0.955). Full receipt-date, reporting-lag, and end-of-window sensitivity results are provided in Supplementary Table S7.

### 3.3. Temporal Change in Occlusion-Associated Coding

**Figure 2:**
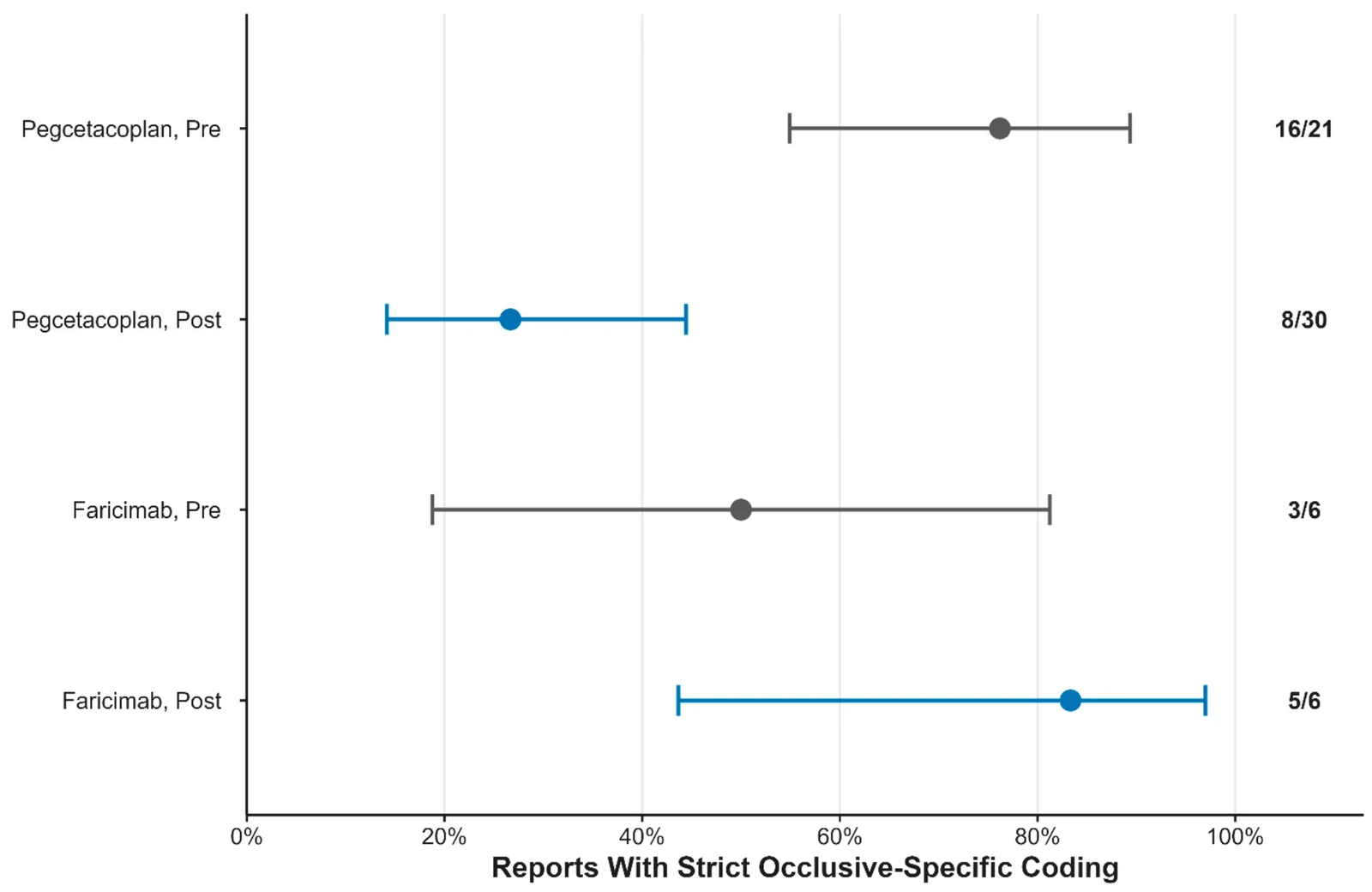
Strict occlusive-specific retinal vasculitis phenotype before and after the August 2023 filter-needle recommendation. Points show the proportion of strict primary-suspect retinal vasculitis reports containing an occlusive-specific vasculitis preferred term, with horizontal error bars representing 95% Wilson confidence intervals. Raw counts are shown as the number of occlusive-specific reports divided by the total number of strict vasculitis reports in each group. The US faricimab comparator included only six reports in each period and should therefore be interpreted cautiously. Classification reflects spontaneous-report terminology and was not independently clinically adjudicated. Results using the expanded occlusion-associated definition are presented in Table 3 and Table S3.

Among pegcetacoplan vasculitis reports, an occlusive-vasculitis preferred term was present in 16 of 21 pre-change reports (76.2%) and eight of 30 post-change reports (26.7%). The odds of occlusive-specific coding were 8.80-fold higher before the change than afterward (95% CI 2.42–31.96; Fisher *p* < 0.001) (Figure 2; Table 3). In the expanded sensitivity analysis, which additionally counted retinal artery, retinal vein, or retinal vascular occlusion when co-reported within a strict vasculitis report, the pre-change proportion remained 16/21 (76.2%) and the post-change proportion increased only to 9/30 (30.0%). The corresponding pre-change versus post-change OR was 7.47 (95% CI 2.09–26.64; *p* = 0.002), indicating that the temporal shift was not dependent on the narrower occlusive-specific preferred-term definition.

Among the US faricimab comparator reports, occlusive-specific coding was present in three of six pre-change reports (50.0%) and five of six post-change reports (83.3%; Fisher *p* = 0.545); these counts were unchanged under the expanded definition. The Firth penalized drug-by-period interaction OR was 0.03 (95% CI 0.002–0.58; *p* = 0.014) using the strict occlusive-specific definition and 0.04 (95% CI 0.002–0.67; *p* = 0.019) using the expanded definition. Because only 12 faricimab reports contributed to the phenotype comparison and classification was based on spontaneous-report terminology rather than clinical adjudication, both interaction analyses were considered exploratory.

### 3.4. Treatment-Context Heterogeneity in US Syfovre Reports

Another anti-VEGF agent was listed in 19 of 79 vasculitis reports (24.1%) and 50 of 571 nonvasculitis reports (8.8%), corresponding to an unadjusted ROR of 3.30 (95% CI 1.83–5.96; Fisher *p* < 0.001). Among the 19 vasculitis reports with anti-VEGF co-reporting, 14 listed aflibercept, two faricimab, two bevacizumab, and one ranibizumab; none listed brolucizumab. Among the 50 anti-VEGF-positive nonvasculitis reports, agent appearances included aflibercept in 29, faricimab in 10, ranibizumab in seven, bevacizumab in five, and brolucizumab in one. Two nonvasculitis reports listed more than one anti-VEGF agent, whereas none of the vasculitis reports did. Agent-specific FAERS drug-role classifications and structured treatment chronology are provided in Supplementary Table S4.

The anti-VEGF estimate remained elevated across several sensitivity analyses. Restricting the exposure definition to anti-VEGF agents coded as suspect or concomitant yielded an ROR of 3.14 (95% CI 1.72–5.74; *p* < 0.001). Excluding faricimab and brolucizumab from the exposure definition yielded an ROR of 3.64 (95% CI 1.95–6.80; *p* < 0.001), and removing all reports that listed either agent yielded an ROR of 3.79 (95% CI 2.02–7.10; *p* < 0.001). Restricting noncases to reports containing ocular adverse events yielded an ROR of 2.84 (95% CI 1.56–5.19; *p* = 0.001), while restriction to reports with retinal or age-related macular degeneration indication context yielded an ROR of 4.31 (95% CI 2.20–8.46; *p* < 0.001).

**Figure 3:**
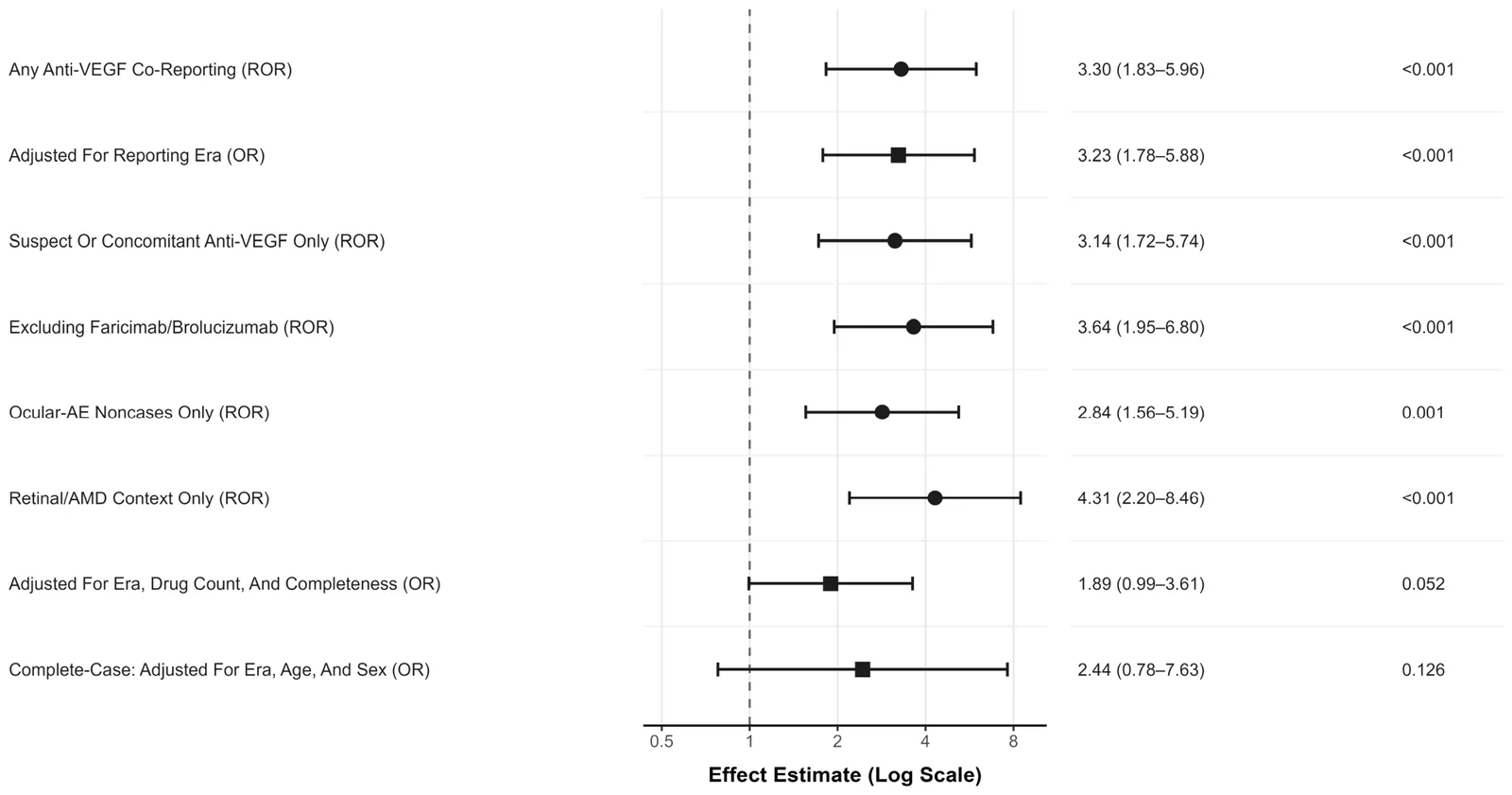
Association between anti-VEGF co-reporting and retinal vasculitis among US primary-suspect Syfovre reports across selected treatment-context and reporting/documentation sensitivity analyses. Points show report-level reporting odds ratios (RORs) or logistic-regression odds ratios (ORs), as indicated in the analysis labels, with 95% confidence intervals. The vertical dashed line denotes an effect estimate of 1, and the *x*-axis is displayed on a logarithmic scale. RORs were derived from two-by-two comparisons within FAERS, whereas ORs were estimated using logistic-regression models. The displayed analyses use substantially overlapping report populations and should be interpreted as sensitivity checks rather than independent replications. These estimates describe disproportionate representation within spontaneous safety reports and should not be interpreted as treated-patient risk estimates, causal effects, or evidence of same-eye or concurrent anti-VEGF exposure. Abbreviations: AE, adverse event; AMD, age-related macular degeneration; anti-VEGF, anti-vascular endothelial growth factor; OR, odds ratio; ROR, reporting odds ratio.

However, vasculitis reports were more information-dense than nonvasculitis reports. Vasculitis reports contained a median of four listed drugs (IQR 2–8) and seven reaction terms, compared with one listed drug (IQR 1–2) and two reaction terms among nonvasculitis reports; the median structured-data completeness score was six versus four. Among nonvasculitis reports, 76.9% contained at least one ocular reaction term; the most frequent reaction preferred terms were vitreous floaters (*n* = 101), visual impairment (*n* = 91), choroidal neovascularisation (*n* = 57), uveitis (*n* = 54), and eye inflammation (*n* = 51). After adjustment for reporting era, total number of listed drugs, and overall report completeness, the anti-VEGF estimate attenuated to OR 1.89 (95% CI 0.99–3.61; *p* = 0.052). This attenuation indicates that differential documentation intensity may contribute to the observed co-reporting association. The previously specified reporting-bias analyses remained elevated, including adjustment for expedited-report status (OR 2.72, 95% CI 1.46–5.07; *p* = 0.002), adjustment for serious-outcome coding (OR 2.58, 95% CI 1.40–4.76; *p* = 0.002), and the exploratory nAMD or choroidal neovascularization-adjusted model (OR 4.03, 95% CI 1.93–8.39; *p* < 0.001). Because these sensitivity analyses were based on substantially overlapping report subsets, they were interpreted as sensitivity checks rather than independent replications of the association (Figure 3; Table 4).

Structured anti-VEGF treatment dates were sufficiently interpretable for only 11 of the 19 anti-VEGF-positive vasculitis reports. The available dates showed mixed treatment sequences, and some reports contained multiple anti-VEGF entries. These data were therefore insufficient to establish whether the listed anti-VEGF therapy preceded, followed, or occurred concurrently with pegcetacoplan exposure in the same eye.

Demographic information was incomplete. Age was available for 151 of 650 reports (23.2%) and sex for 225 (34.6%). The complete-case demographic sensitivity model included 149 reports, of which 27 were vasculitis reports, and simultaneously included anti-VEGF co-reporting, reporting era, age, and sex. Anti-VEGF co-reporting had an OR of 2.44 (95% CI 0.78–7.63; *p* = 0.126), while post-August 2023 reporting era had an OR of 0.34 (95% CI 0.14–0.84; *p* = 0.020). The OR was 0.66 per 10-year increase in age (95% CI 0.42–1.02; *p* = 0.063) and 0.90 for female versus male sex (95% CI 0.36–2.28; *p* = 0.826). These estimates were imprecise because demographic completeness substantially reduced the analytic sample. Structured bilateral treatment was documented in 24 reports but in no vasculitis cases, producing sparse-data separation; an adjusted bilateral-treatment model was therefore not reported.

## 4. Discussion

This study provides three complementary observations about the postmarketing safety signal for intravitreal pegcetacoplan. First, retinal vasculitis reporting per estimated vial volume fell markedly after the August 2023 filter-needle recommendation, while faricimab reporting also changed over the same calendar period. The descriptive comparison between products was imprecise and did not establish that their proportional temporal changes were similar. Second, the composition of pegcetacoplan vasculitis reports changed substantially, with occlusive-coded events decreasing from approximately three-quarters of pre-change reports to approximately one-quarter of post-change reports. Third, within all US primary-suspect Syfovre reports, retinal vasculitis was disproportionately represented when another anti-VEGF agent was also listed; however, the association attenuated after adjustment for medication count and report completeness, while remaining elevated in several other reporting-bias sensitivity analyses.

These findings should be interpreted in the context of prior pharmacovigilance work. Previous FAERS studies have characterized ocular adverse events associated with complement inhibitors and, more recently, retinal vasculitis reporting across multiple intravitreal anti-VEGF agents and complement inhibitors [11,12]. The present study therefore does not claim retinal vasculitis as a newly identified pegcetacoplan safety signal. Its incremental contribution is the longitudinal examination of reporting before and after the August 2023 filter-needle recommendation, the assessment of temporal changes in occlusion-associated coding using both strict and expanded definitions, and the evaluation of anti-VEGF co-reporting within the broader Syfovre safety-report population.

The temporal findings argue for caution in attributing the decline to the filter-needle recommendation itself. The crude pegcetacoplan reporting-rate fell substantially between the defined periods, while faricimab reporting also changed over the same calendar period. However, faricimab cannot be assumed to represent only background reporting behavior. In November 2023, near the beginning of the post-change period, Genentech issued an Important Drug Warning concerning retinal vasculitis and/or retinal vascular occlusion following postmarketing reports with faricimab [20]. Faricimab also has its own pharmacovigilance and clinical literature relevant to retinal inflammatory events. He et al. reported a FAERS disproportionality analysis of faricimab adverse-event reporting [21], and Bruening et al. described clinical cases of inflammation and occlusive retinal vasculitis following faricimab [22]. Faricimab should therefore not be interpreted as a safety-neutral comparator. The comparison also does not imply a shared biological mechanism. Intraocular inflammation associated with different intravitreal agents may arise through different drug-, formulation-, immune-, or delivery-related pathways [8,22]. Our analysis does not test those mechanisms and uses faricimab only to describe how retinal vasculitis reporting changed over the same calendar period. This communication could itself have altered reporting for the comparator. In addition, only two pre-change quarters were available, one of which included the pegcetacoplan launch period, so comparable underlying pre-change trends between the products could not be established. The RRR should therefore be interpreted as a descriptive contemporaneous contrast rather than an adjustment for general reporting attenuation. Its wide confidence interval permits substantial relative differences in either direction and does not demonstrate that the two products experienced similar temporal changes.

Exploratory quarterly modeling likewise did not provide a simple intervention-effect interpretation. A level-change model did not identify a clear differential post-recommendation level change, whereas a segmented model suggested a steeper subsequent decline in pegcetacoplan reporting relative to faricimab. These estimates depend on modeling the trajectory from only two pre-change quarters and very small comparator event counts. The segmented coefficients should therefore not be interpreted as evidence that the filter-needle recommendation caused a change in reporting trajectory. Rather, they illustrate that the observed temporal pattern is more complex than a single pooled pre-change versus post-change contrast and is sensitive to how calendar time is modeled.

A separate limitation is the changing composition of the exposure denominator as treatment becomes established. In the initial ASRS ReST series, all 14 affected eyes developed vasculitis after their first pegcetacoplan injection [6]. Our quarterly denominator captures total vial volume and cannot distinguish treatment initiations from repeat injections. As repeat treatment becomes a larger component of total vial use, reports per total vials could decline even if the underlying event probability associated with treatment initiation remained unchanged. Quarter-specific numbers of first injections were unavailable, preventing direct evaluation of this possibility. The faricimab comparison does not resolve this limitation because the proportion of first versus repeat injections may evolve differently between the two products.

These considerations further limit causal interpretation of the August 2023 filter-needle recommendation. Case-level filter-needle exposure was unavailable in FAERS, and the recommendation defines only a calendar boundary rather than a verified patient-level transition in device exposure. Moreover, in the ASRS ReST series, filter-needle gauge was known for only four affected eyes: two involved 19-gauge filter needles and two involved 18-gauge filter needles [6]. Thus, the early clinical series did not uniquely associate reported vasculitis with one needle gauge. The temporal analysis cannot establish immediate depletion of older inventory, individual needle exposure, or a causal mechanism [8].

The phenotype analysis provides a complementary description of how vasculitis reports were coded over time. The early ASRS ReST series identified occlusive retinal vasculopathy in 11 of 14 affected eyes (79%), closely resembling the 76.2% occlusive-specific proportion in our pre-change FAERS cohort [6]. In contrast, 26.7% of post-change pegcetacoplan reports contained an occlusive-vasculitis preferred term. This pattern was largely unchanged when the phenotype definition was broadened to include retinal artery, retinal vein, or retinal vascular occlusion reported alongside strict retinal vasculitis: the post-change proportion increased only to 30.0%, compared with 76.2% before the change. Thus, the observed shift was not driven solely by use of the narrower occlusive-vasculitis preferred terms. However, the broader sensitivity analysis does not eliminate observation or classification bias. Increased awareness of pegcetacoplan-associated vasculitis and evolving coding practices could change which MedDRA terms were selected over time, even for clinically similar events.

FAERS terminology cannot establish the clinical presence, anatomical extent, or severity of retinal vascular occlusion, and preferred-term coding is not equivalent to an adjudicated angiographic phenotype. The within-pegcetacoplan shift should therefore be interpreted as a change in the terminology and composition of reported vasculitis cases rather than evidence that the biological severity of events decreased. The drug-by-period interaction was similar under the strict and expanded definitions but remains exploratory because only 12 faricimab reports contributed to the comparison and should not be interpreted as confirmatory evidence of a differential clinical phenotype between products.

Anti-VEGF co-reporting was more frequent among Syfovre vasculitis reports than among nonvasculitis reports, and the association persisted when exposure was restricted by drug role, when faricimab and brolucizumab were excluded, and when the comparison was restricted to reports with ocular or retinal disease context. This analysis does not imply that pegcetacoplan and anti-VEGF agents produce inflammation through the same biological pathway. The agent-specific distribution was dominated by aflibercept rather than by anti-VEGF agents with their own prominent inflammatory safety signals. These findings make it less likely that the association is explained solely by the inclusion of faricimab or brolucizumab. The limited structured chronology further argues against interpreting co-reporting as concurrent exposure. Therapy-start and event dates were jointly interpretable for only 11 of 19 anti-VEGF-positive vasculitis reports, and the available sequences included treatment starts both before and after the recorded event date. Co-reporting may therefore represent prior therapy, ongoing therapy, treatment initiated after exudation or another retinal event, or medication history. FAERS cannot determine whether an anti-VEGF agent was administered in the affected eye or was active at the time vasculitis developed.

At the same time, the association attenuated substantially after adjustment for the number of documented medications and overall report completeness. Vasculitis reports were more information-dense than nonvasculitis reports, creating greater opportunity for prior or concomitant medications to be recorded. The co-reporting signal therefore may partly reflect differential documentation rather than a biological interaction. FAERS also could not reliably establish treatment chronology, active co-administration, same-eye exposure, or injection sequence. Accordingly, these analyses describe treatment-context co-reporting rather than evidence that anti-VEGF therapy increases the risk of pegcetacoplan-associated vasculitis.

Several noncausal explanations remain plausible. Patients receiving anti-VEGF therapy may have coexisting or incident neovascular disease, greater cumulative injection exposure, closer surveillance, or more detailed medication documentation. New-onset exudation has been reported during pegcetacoplan treatment [13], and real-world studies confirm that complement inhibition and anti-VEGF management commonly coexist [14,15]. Anti-VEGF co-reporting may therefore be a marker of retinal disease complexity and reporting intensity rather than an independent safety modifier. Prospective eye-level data with treatment sequence, laterality, injection burden, and same-eye exposure are required to determine whether any biological relationship exists.

This distinction is central to the personalized-medicine relevance of the study. Personalized medication safety does not require that a spontaneous-reporting database predict an individual adverse event. Rather, pharmacovigilance can identify heterogeneity and uncertainty that warrant better prospective evidence. For GA, treatment selection incorporates lesion characteristics, expected progression, treatment frequency, visual status, and safety [3,4,5]. Our findings add postmarketing evidence that reported safety patterns vary over time, by reported phenotype, and by treatment context. However, the anti-VEGF co-reporting analysis is not sufficiently causal or eye-specific to justify different counseling or treatment decisions for patients receiving anti-VEGF therapy. Its principal value is to identify a clinically relevant subgroup for prospective study.

The study has several strengths. We re-linked cases across the FAERS relational structure, retained the latest case version, applied a strict retinal vasculitis preferred-term definition, excluded retinal vascular-occlusion reports lacking vasculitis terminology, and required primary-suspect target-drug status in the primary analysis. We corrected geographic numerator-denominator mismatch by restricting faricimab reports to US reporters when US sales were used for exposure estimation. The Syfovre case–noncase analysis excluded systemic pegcetacoplan and evaluated the complete US primary-suspect reporting population rather than only selected vasculitis reports. Finally, the anti-VEGF association was evaluated across multiple sensitivity analyses addressing reporting characteristics, treatment context, and documentation intensity rather than relying on a single crude disproportionality estimate.

The limitations are substantial and should govern interpretation. FAERS is subject to underreporting, stimulated reporting, duplicate submissions, variable completeness, differential publicity, and uncertain causal attribution [9,10,16,23]. Retaining only the latest version of each FAERS case identifier removes identifiable follow-up versions, and our cross-identifier screen did not flag candidate duplicate reports. Nevertheless, distinct case identifiers may still represent the same patient or clinical event when identifying information is incomplete, so report counts should not be interpreted as verified numbers of unique patients or eyes. FDA similarly cautions that spontaneous reports may be duplicate or incomplete, do not establish causation, may contain unverified information, and cannot be used to estimate incidence [23]. Primary-suspect status does not prove causality. Reporter country is an imperfect proxy for treatment geography. Manufacturer-distributed vials and revenue-derived unit estimates are imperfect proxies for administered injections and do not distinguish treatment initiation from repeat dosing. The Q1 2023 pegcetacoplan denominator is itself a conservative lower-bound estimate because the manufacturer reported more than 6000 commercial vials and more than 3400 sample vials rather than exact counts. The faricimab denominator depends on the conversion of reported US sales to estimated vial volume using annual currency conversion and a fixed launch wholesale acquisition cost, and therefore does not capture discounts, rebates, subsequent price changes, or other changes in net price. Time-varying error in either denominator could alter the estimated pre-change versus post-change reporting ratios, as reflected by the denominator stress tests. Because the two products’ exposure proxies were reconstructed using different source types and assumptions, their absolute reporting rates should not be interpreted as directly comparable measures of clinical incidence. Faricimab also differs from pegcetacoplan in indication, launch timing, treatment sequence, public attention, and baseline susceptibility to vasculitis reporting, and its November 2023 retinal vasculitis safety communication could have independently affected reporting. Only two pre-change quarters were available, precluding assessment of comparable underlying pre-change trends. Faricimab should therefore be viewed as a descriptive contemporaneous reporting comparator rather than a control group or adjustment for general reporting attenuation. Comparator event counts were small, further contributing to the wide uncertainty around the RRRs.

Additional limitations apply to the secondary analyses. Individual filter-needle exposure was unknown, so pre-change and post-change periods may misclassify actual kit use. Occlusive status was inferred from MedDRA preferred terms without independent clinical adjudication. The primary temporal analysis required a quarter-assignable clinical event date, and availability differed substantially between products and across receipt periods, particularly for faricimab. Reports with and without quarter-assignable event dates were similar with respect to coded seriousness and strict occlusive-vasculitis coding, but pegcetacoplan report-version distributions differed, and selection related to unmeasured report characteristics cannot be excluded. The complementary initial-receipt-date analysis included all reports but indexed reporting activity rather than clinical event timing and produced a different descriptive temporal pattern with wide confidence intervals. Reporting lags were also substantial and variable. Analyses excluding the final six or 12 months remained imprecise and cannot fully resolve delayed ascertainment near the 31 December 2025 cutoff. Differential event-date availability and reporting delay therefore remain important limitations of the comparator-adjusted temporal analysis. The case–noncase ROR describes the composition of Syfovre adverse-event reports, not risk among treated patients. Although sensitivity analyses examined anti-VEGF drug role and treatment context, FAERS did not reliably establish chronology, active co-administration, laterality, injection count, or same-eye exposure. The association also attenuated after accounting for medication count and report completeness, indicating potential confounding by documentation intensity, disease complexity, treatment burden, and surveillance. Public FAERS files lack detailed narratives, imaging, visual acuity trajectories, and definitive treatment sequencing. Age and sex were frequently missing. Finally, secondary and exploratory comparisons were not adjusted for multiplicity and require external validation.

Taken together, these results characterize changes in postmarketing safety reporting rather than changes in patient-level clinical risk. Pegcetacoplan reporting per estimated vial volume declined over time, but limitations of the comparator, changes in the composition of vial exposure, and denominator uncertainty prevent attributing that decline to the filter-needle recommendation or interpreting it as evidence of improved clinical safety. The reported vasculitis phenotype shifted away from occlusive-specific terminology, but this does not establish that clinically occlusive events became less frequent among treated eyes. Anti-VEGF co-reporting was more frequent among vasculitis reports, but attenuation after adjustment for documentation intensity and the absence of reliable eye-level treatment chronology preclude subgroup-specific risk inference. These observations identify priorities for prospective eye-level study but do not provide a basis for changing individual patient counseling.

## 5. Conclusions

Retinal vasculitis reporting with intravitreal pegcetacoplan declined markedly after the August 2023 filter-needle recommendation. The descriptive faricimab comparison was imprecise and could not determine whether the proportional temporal change differed between products. The reported pegcetacoplan phenotype shifted substantially away from occlusive-specific coding, but FAERS terminology cannot establish that clinically occlusive vasculitis became less frequent among treated eyes. Within US primary-suspect Syfovre reports, another anti-VEGF agent was more frequently co-reported with vasculitis than with nonvasculitis reports, but this association attenuated after adjustment for medication count and report completeness. These findings describe temporal, phenotypic, and treatment-context heterogeneity in spontaneous safety reporting. They should not be interpreted as evidence that the filter-needle recommendation improved clinical safety, that vasculitis severity decreased, or that patients receiving anti-VEGF therapy have greater clinical risk. Prospective eye-level studies with verified injection chronology, laterality, imaging phenotype, and treatment denominators are needed to determine whether these reporting patterns correspond to differences in clinically adjudicated outcomes.

## Figures and Tables

**Table 1:** Analytic cohorts and geography matching.

Measure	Pegcetacoplan/Syfovre	Faricimab Comparator
US strict primary-suspect retinal vasculitis reports identified in study window	79	45
Reports with quarter-assignable event dates for temporal analysis	58/79 (73.4%)	14/45 (31.1%)
Pre-change reports used in temporal analysis	21	6
Q3 2023 transition-quarter reports	7	2
Post-change reports used in temporal analysis	30	6
US primary-suspect case–noncase cohort	650	Not applicable
Strict vasculitis cases within case–noncase cohort	79	Not applicable
Nonvasculitis reports within case–noncase cohort	571	Not applicable
Occlusive-coded reports among case–noncase vasculitis cases	42/79 (53.2%)	Not applicable
Age available in case–noncase cohort	151/650 (23.2%)	Not applicable
Sex available in case–noncase cohort	225/650 (34.6%)	Not applicable

Note: The temporal analysis required a quarter-assignable event date. The US Syfovre case–noncase cohort used initial FDA receipt date and therefore included strict vasculitis reports without quarter-assignable event dates. Only US-reporter faricimab reports were used with the US sales-derived exposure denominator. Abbreviations: Q3, third quarter.

**Table 2:** Temporal retinal vasculitis reporting-rate analyses.

Analysis	Pegcetacoplan Reporting RR (95% CI)	US Faricimab Reporting RR (95% CI)	Ratio of Reporting RRs (95% CI)	*p* Value
Primary: Strict vasculitis, target-drug primary suspect	0.09 (0.05–0.16)	0.12 (0.04–0.36)	0.79 (0.22–2.78)	0.711
Sensitivity: Primary or secondary suspect	0.09 (0.05–0.16)	0.16 (0.05–0.45)	0.59 (0.18–1.96)	0.389
Sensitivity: Q1 2023 excluded	0.08 (0.04–0.13)	0.13 (0.03–0.51)	0.59 (0.13–2.64)	0.493
Sensitivity: Post-change period begins Q1 2024	0.06 (0.03–0.11)	0.11 (0.03–0.35)	0.56 (0.15–2.15)	0.399

Note: RR indicates the reporting-rate ratio comparing post-change with pre-change calendar periods. The p value tests the ratio of reporting RRs. Q3 2023 was excluded as the transition quarter. Reporting rates use estimated vial denominators and should not be interpreted as clinical incidence. Abbreviations: CI, confidence interval; Q1, first quarter; RR, reporting-rate ratio; and US, the United States.

**Table 3:** Strict and expanded occlusion-associated retinal vasculitis before and after the August 2023 filter-needle recommendation.

Definition	Cohort/Comparison	Pre-Change	Post-Change	Estimate (95% CI)	*p* Value
Strict occlusive-specific PT	Pegcetacoplan	16/21 (76.2%)	8/30 (26.7%)	Pre vs. post OR 8.80 (2.42–31.96)	<0.001
Strict occlusive-specific PT	US faricimab	3/6 (50.0%)	5/6 (83.3%)	Pre vs. post OR 0.20 (0.01–2.91)	0.545
Strict occlusive-specific PT	Drug-by-period interaction, Firth	Not applicable	Not applicable	Interaction OR 0.03 (0.002–0.58)	0.014
Expanded occlusion-associated definition	Pegcetacoplan	16/21 (76.2%)	9/30 (30.0%)	Pre vs. post OR 7.47 (2.09–26.64)	0.002
Expanded occlusion-associated definition	US faricimab	3/6 (50.0%)	5/6 (83.3%)	Pre vs. post OR 0.20 (0.01–2.91)	0.545
Expanded occlusion-associated definition	Drug-by-period interaction, Firth	Not applicable	Not applicable	Interaction OR 0.04 (0.002–0.67)	0.019

Note: The strict phenotype required an occlusive-vasculitis preferred term. The expanded definition additionally classified a strict retinal vasculitis report as occlusion-associated when retinal artery occlusion, retinal vein occlusion, or retinal vascular occlusion was also reported. Pre-change versus post-change *p* values are from two-sided Fisher exact tests. Drug-by-period interactions were estimated using Firth penalized logistic regression because faricimab cells were sparse; interaction confidence intervals are approximate Wald intervals and *p* values are penalized likelihood-ratio *p* values. Interaction analyses are exploratory. Abbreviations: CI, confidence interval; OR, odds ratio; PT, preferred term; and US, the United States.

**Table 4:** Robustness of the association between anti-VEGF co-reporting and retinal vasculitis within US primary-suspect Syfovre reports.

Analysis	N (Vasculitis Cases)	Measure	Effect Estimate (95% CI)	*p* Value
Unadjusted any anti-VEGF co-reporting	650 (79)	ROR	3.30 (1.83–5.96)	<0.001
Primary model, adjusted for reporting era	650 (79)	OR	3.23 (1.78–5.88)	<0.001
Anti-VEGF coded suspect or concomitant only	650 (79)	ROR	3.14 (1.72–5.74)	<0.001
Exposure excludes faricimab and brolucizumab	650 (79)	ROR	3.64 (1.95–6.80)	<0.001
Drop all reports listing faricimab or brolucizumab	637 (77)	ROR	3.79 (2.02–7.10)	<0.001
Ocular-adverse-event noncases only	518 (79)	ROR	2.84 (1.56–5.19)	0.001
Retinal/AMD indication-context reports only	407 (54)	ROR	4.31 (2.20–8.46)	<0.001
Adjusted for reporting era, drug count, and report completeness	650 (79)	OR	1.89 (0.99–3.61)	0.052
Complete-case model adjusted for era, age, and sex	149 (27)	OR	2.44 (0.78–7.63)	0.126
Adjusted for expedited-report status	650 (79)	OR	2.72 (1.46–5.07)	0.002
Adjusted for serious-outcome coding	650 (79)	OR	2.58 (1.40–4.76)	0.002
Restricted to serious-outcome reports	430 (76)	OR	2.67 (1.44–4.97)	0.002
Restricted to expedited reports	330 (67)	OR	2.42 (1.22–4.77)	0.011
Restricted to expedited or direct reports	357 (71)	OR	2.55 (1.31–4.96)	0.006
Exploratory model with nAMD/CNV indication adjustment	650 (79)	OR	4.03 (1.93–8.39)	<0.001

Note: RORs are unadjusted FAERS reporting odds ratios from two-bytwo comparisons; ORs are logistic-regression estimates. These analyses describe the composition of spontaneous safety reports and do not estimate treated-patient risk. Anti-VEGF co-reporting does not establish chronology, same-eye treatment, concurrent exposure, or causality. Sensitivity analyses use substantially overlapping report populations and should not be interpreted as independent replications. Abbreviations: AMD, age-related macular degeneration; anti-VEGF, anti-vascular endothelial growth factor; CI, confidence interval; CNV, choroidal neovascularization; nAMD, neovascular age-related macular degeneration; OR, odds ratio; ROR, reporting odds ratio.

## Data Availability

The source FAERS data are publicly available from the US Food and Drug Administration. Exposure-denominator source data are publicly available through manufacturer financial disclosures.

## Supplementary Materials

The following supporting information can be downloaded at: https://www.mdpi.com/article/10.3390/jpm16100533/s1, Supplementary Methods; Table S1, duplicate and cohort-overlap audit; Table S2, quarterly event counts, exposure denominators, reporting rates, and denominator audit; Table S3, strict and expanded occlusion-associated phenotype analyses; Table S4, anti-VEGF agent, FAERS drug-role, and structured treatment chronology; Table S5, Temporal robustness analyses: denominator stress tests and exploratory quarterly Poisson models; Table S6, event-date completeness and dated-versus-undated report characteristics; Table S7, receipt-date sensitivity, reporting lag, and end-of-window analyses; Table S8, treatment-context sensitivity and report-completeness analyses.

## Abbreviations

AE	adverse event
AMD	age-related macular degeneration
anti-VEGF	anti-vascular endothelial growth factor
ASRS	American Society of Retina Specialists
CI	confidence interval
CNV	choroidal neovascularization
FAERS	US Food and Drug Administration Adverse Event Reporting System
FDA	US Food and Drug Administration
GA	geographic atrophy
IQR	interquartile range
MedDRA	Medical Dictionary for Regulatory Activities
nAMD	neovascular age-related macular degeneration
OR	odds ratio
PT	preferred term
Q1	first quarter
Q2	second quarter
Q3	third quarter
Q4	fourth quarter
ReST	Research and Safety in Therapeutics
ROR	reporting odds ratio
RR	reporting-rate ratio
RRRs	ratio of reporting-rate ratios
US	The United States
USD	US dollar
