# Supplementary material for: Temporal, Phenotypic, and Treatment-Context Heterogeneity in Retinal Vasculitis Reporting with Pegcetacoplan: A United States Pharmacovigilance Study
Source: J Pers Med. 2026 Oct 16;16(10):533. doi: 10.3390/jpm16100533 (PMC13705734; doi:10.3390/jpm16100533)
Supplement: Supplementary file 1 [file jpm-16-00533-s001.zip › jpm-4555396-supplementary.pdf]

Supplementary Materials

Temporal, Phenotypic, and Treatment-Context Heterogeneity in Retinal Vasculitis Reporting with Pegcetacoplan: A United States Pharmacovigilance Study

Siddharth Gandhi, Michael Balas, Patrick Wang, and Parnian Arjmand

Contents

Supplementary Methods
Supplementary Table S1. Duplicate and cohort-overlap audit
Supplementary Table S2. Quarterly event counts, exposure denominators, reporting rates, and denominator audit
Supplementary Table S3. Strict and expanded occlusion-associated phenotype analyses
Supplementary Table S4. Anti-VEGF agent, FAERS drug-role, and structured treatment chronology
Supplementary Table S5. Temporal robustness analyses: denominator stress tests and exploratory quarterly Poisson models
Supplementary Table S6. Event-date completeness and dated-versus-undated report characteristics
Supplementary Table S7. Receipt-date sensitivity, reporting lag, and end-of-window analyses
Supplementary Table S8. Treatment-context sensitivity and report-completeness analyses

Supplementary Methods

Duplicate audit

Same-identifier follow-up versions were collapsed to the latest report version. A cross-identifier screen flagged pairs sharing the same manufacturer report number or matching on sex, age, clinical event date, and reporter country with at least 50% overlap in reaction terms. The screen was used as an audit rather than as an automatic exclusion rule.

Exposure-denominator audit

Pegcetacoplan exposure was reconstructed from manufacturer-reported commercial and sample or free-goods vial distributions. For Q1 2023, 9,400 vials were used as a conservative lower-bound proxy based on more than 6,000 commercial and more than 3,400 sample vials. Faricimab US vial volume was estimated from Roche US sales converted using annual average CHF/USD exchange rates and divided by the launch wholesale acquisition cost of USD 2,190 per treatment. Exposure quantities are proxies rather than verified administered injections.

Phenotype sensitivity analysis

The strict occlusive-coded phenotype required a preferred term of retinal occlusive vasculitis, haemorrhagic occlusive retinal vasculitis, or hemorrhagic occlusive retinal vasculitis. The expanded occlusion-associated definition additionally counted retinal artery occlusion, retinal vein occlusion, or retinal vascular occlusion when these occurred within a report that already met the strict retinal vasculitis case definition.

Anti-VEGF treatment context

Anti-VEGF agents were summarized individually and by FAERS drug role. Structured therapy dates were summarized descriptively and were not assumed to establish same-eye or active concurrent exposure.

Temporal robustness

Deterministic denominator stress tests assessed sensitivity to time-varying denominator error. Exploratory Poisson regression models used quarterly event counts with log estimated vial exposure as an offset. Q3 2023 was excluded as the transition quarter. A level-change model included drug, calendar time, post-recommendation period, and a drug-by-post-period interaction. A segmented model additionally included time after the transition quarter and its interaction with drug. Heteroskedasticity-consistent (HC0) sandwich standard errors were used.

Event-date completeness

A clinical event date was considered quarter-assignable when at least year and month were available in the primary temporal date source. Dated and undated strict US primary-suspect retinal vasculitis reports were compared within each drug by receipt period, coded serious outcome, strict and expanded occlusion-associated coding, report-version number, reporter occupation, and FAERS report type.

Receipt-date sensitivity and reporting lag

As a complementary analysis, all strict US primary-suspect retinal vasculitis reports were indexed by initial FDA receipt quarter using the same pre-change, transition, and post-change calendar definitions as the primary analysis. Reporting lag was summarized in quarters and, when exact clinical event dates were available, in days from event date to initial FDA receipt.

Treatment-context sensitivity

Sensitivity analyses restricted anti-VEGF exposure by drug role, excluded agents with their own inflammatory safety signals, restricted the noncase population to ocular adverse-event reports, restricted to retinal or age-related macular degeneration indication context, and adjusted for documentation intensity.

Supplementary Table S1. Duplicate and cohort-overlap audit

Target-cohort overlap and cross-identifier duplicate screen

Audit item	Result
Pegcetacoplan strict US primary-suspect retinal vasculitis reports	79
Faricimab strict US primary-suspect retinal vasculitis reports	45
Shared FAERS case identifiers between target-drug cohorts	0
Potential cross-identifier duplicate pairs flagged within drug cohorts	0

No shared case identifiers were identified between the pegcetacoplan and faricimab target-drug cohorts. The prespecified cross-identifier duplicate screen identified no candidate pairs. Because FAERS fields can be incomplete or inconsistently recorded, unrecognized duplication cannot be excluded; counts represent reports rather than verified unique patients or eyes.

Supplementary Table S2. Quarterly event counts, exposure denominators, reporting rates, and denominator audit

Quarterly event counts, estimated vial denominators, and reporting rates with exact Poisson 95% confidence intervals

Quarter	Peg reports	Peg estimated vials	Peg rate/10,000 (95% CI)	Far reports	Far estimated vials	Far rate/10,000 (95% CI)
Q1 2023	0	9400	0.000 (0.000-3.924)	3	182500	0.164 (0.034-0.480)
Q2 2023	21	42000	5.000 (3.095-7.643)	3	216900	0.138 (0.029-0.404)
Q3 2023	7	47000	1.489 (0.599-3.069)	2	269600	0.074 (0.009-0.268)
Q4 2023	12	62000	1.935 (1.000-3.381)	1	301100	0.033 (0.001-0.185)
Q1 2024	7	77000	0.909 (0.366-1.873)	1	338400	0.030 (0.001-0.165)
Q2 2024	4	84000	0.476 (0.130-1.219)	1	375300	0.027 (0.001-0.148)
Q3 2024	3	88500	0.339 (0.070-0.991)	1	403400	0.025 (0.001-0.138)
Q4 2024	3	94000	0.319 (0.066-0.933)	1	413300	0.024 (0.001-0.135)
Q1 2025	0	92000	0.000 (0.000-0.401)	0	396700	0.000 (0.000-0.093)
Q2 2025	1	95000	0.105 (0.003-0.586)	1	404400	0.025 (0.001-0.138)
Q3 2025	0	101000	0.000 (0.000-0.365)	0	380700	0.000 (0.000-0.097)
Q4 2025	0	102000	0.000 (0.000-0.362)	0	396700	0.000 (0.000-0.093)

Denominator reconstruction audit by quarter

Quarter	Syfovre commercial component	Syfovre sample/free component	Syfovre final vials	Syfovre source document	Vabysmo US sales (CHF millions)	CHF/USD annual average	Vabysmo estimated vials
Q1 2023	6000	3400	9400	Apellis Q1 2023 financial results presentation	360	1.11	182500
Q2 2023	31000	11000	42000	Apellis Q2 2023 financial results	428	1.11	216900
Q3 2023	37000	10000	47000	Apellis Q3 2023 financial results	532	1.11	269600
Q4 2023	55000	6400	62000	Apellis Q4/FY 2023 financial results	594	1.11	301100
Q1 2024	72000	5000	77000	Apellis Q1 2024 financial results	650	1.14	338400
Q2 2024	79000	5000	84000	Apellis Q2 2024 financial results	721	1.14	375300
Q3 2024	84500	4000	88500	Apellis Q3 2024 financial results	775	1.14	403400
Q4 2024	89000	4600	94000	Apellis Q4/FY 2024 financial results	794	1.14	413300
Q1 2025	82000	10000	92000	Apellis Q1 2025 financial results	718	1.21	396700

Q2 2025	82000	13000	95000	Apellis Q2 2025 financial results	732	1.21	404400
Q3 2025	86000	15000	101000	Apellis Q3 2025 financial results	689	1.21	380700
Q4 2025	89000	13000	102000	Apellis Q4/FY 2025 financial results	718	1.21	396700

For Q1 2023, the manufacturer reported more than 6,000 commercial vials and more than 3,400 sample vials; the analysis used 9,400 as a conservative lower-bound exposure proxy. Faricimab vial estimates used Roche US sales converted to USD using the annual average CHF/USD rate and divided by a fixed launch WAC of USD 2,190 per treatment. Exposure estimates are proxies and their uncertainty is not propagated into the reported confidence intervals. Quarter-specific source documents are identified in the denominator audit above; the main manuscript cites the manufacturer financial disclosure and pricing sources used for exposure reconstruction.

Supplementary Table S3. Strict and expanded occlusion-associated phenotype analyses

Strict and expanded occlusion-associated phenotype proportions

Definition	Cohort	Period	Occlusion-associated n/N (%)	Wilson 95% CI (%)
Strict occlusive-specific PT	Pegcetacoplan	Pre	16/21 (76.2%)	54.9-89.4
Strict occlusive-specific PT	Pegcetacoplan	Post	8/30 (26.7%)	14.2-44.4
Strict occlusive-specific PT	Faricimab	Pre	3/6 (50.0%)	18.8-81.2
Strict occlusive-specific PT	Faricimab	Post	5/6 (83.3%)	43.6-97.0
Expanded occlusion-associated	Pegcetacoplan	Pre	16/21 (76.2%)	54.9-89.4
Expanded occlusion-associated	Pegcetacoplan	Post	9/30 (30.0%)	16.7-47.9
Expanded occlusion-associated	Faricimab	Pre	3/6 (50.0%)	18.8-81.2
Expanded occlusion-associated	Faricimab	Post	5/6 (83.3%)	43.6-97.0

Pre-change versus post-change phenotype comparisons

Definition	Cohort	Pre-change	Post-change	Pre vs post OR (95% CI)	Fisher p value
Strict occlusive-specific PT	Pegcetacoplan	16/21	8/30	8.80 (2.42-31.96)	<0.001
Strict occlusive-specific PT	Faricimab	3/6	5/6	0.20 (0.01-2.91)	0.545
Expanded occlusion-associated	Pegcetacoplan	16/21	9/30	7.47 (2.09-26.64)	0.002
Expanded occlusion-associated	Faricimab	3/6	5/6	0.20 (0.01-2.91)	0.545

Penalized drug-by-period phenotype interactions

Definition	Method	Interaction OR (95% CI)	Penalized LR p value	Total N	Faricimab N
Strict occlusive-specific PT	Firth penalized logistic regression	0.034 (0.002-0.579)	0.014	63	12
Expanded occlusion-associated	Firth penalized logistic regression	0.040 (0.002-0.673)	0.019	63	12

Firth penalized logistic regression was used because comparator cells were sparse. Confidence intervals are approximate Wald intervals. These interaction analyses are exploratory.

Exact preferred-term combinations among temporally assignable strict retinal vasculitis reports

Cohort	Period	Relevant preferred-term combination	N
Faricimab	Post	retinal occlusive vasculitis	4

Faricimab	Post	haemorrhagic occlusive retinal vasculitis	1
Faricimab	Post	retinal vasculitis	1
Faricimab	Pre	retinal occlusive vasculitis	3
Faricimab	Pre	retinal vasculitis	3
Pegcetacoplan	Post	retinal vasculitis	21
Pegcetacoplan	Post	retinal occlusive vasculitis	5
Pegcetacoplan	Post	haemorrhagic occlusive retinal vasculitis	2
Pegcetacoplan	Post	retinal occlusive vasculitis + retinal vein occlusion	1
Pegcetacoplan	Post	retinal vascular occlusion + retinal vasculitis	1
Pegcetacoplan	Pre	retinal occlusive vasculitis	7
Pegcetacoplan	Pre	haemorrhagic occlusive retinal vasculitis	5
Pegcetacoplan	Pre	retinal vasculitis	5
Pegcetacoplan	Pre	retinal occlusive vasculitis + retinal vasculitis	2
Pegcetacoplan	Pre	haemorrhagic occlusive retinal vasculitis + retinal occlusive vasculitis	1
Pegcetacoplan	Pre	retinal artery occlusion + retinal occlusive vasculitis	1

Preferred-term mapping used for strict and expanded phenotype definitions

Preferred term	Role in phenotype definition	MedDRA version handling
haemorrhagic occlusive retinal vasculitis	Strict vasculitis qualifying PT	Not encoded as a case-level field in the public FAERS ASCII data used here; PT strings analyzed as supplied in REAC.pt
hemorrhagic occlusive retinal vasculitis	Strict vasculitis qualifying PT	Not encoded as a case-level field in the public FAERS ASCII data used here; PT strings analyzed as supplied in REAC.pt
retinal artery occlusion	Expanded occlusion-associated PT	Not encoded as a case-level field in the public FAERS ASCII data used here; PT strings analyzed as supplied in REAC.pt
retinal occlusive vasculitis	Strict vasculitis qualifying PT	Not encoded as a case-level field in the public FAERS ASCII data used here; PT strings analyzed as supplied in REAC.pt
retinal vascular occlusion	Expanded occlusion-associated PT	Not encoded as a case-level field in the public FAERS ASCII data used here; PT strings analyzed as supplied in REAC.pt

retinal vasculitis	Strict vasculitis qualifying PT	Not encoded as a case-level field in the public FAERS ASCII data used here; PT strings analyzed as supplied in REAC.pt
retinal vein occlusion	Expanded occlusion-associated PT	Not encoded as a case-level field in the public FAERS ASCII data used here; PT strings analyzed as supplied in REAC.pt

The public FAERS ASCII data used in this analysis do not encode a case-level MedDRA version. Preferred-term strings were analyzed as supplied in the REAC table.

Supplementary Table S4. Anti-VEGF agent, FAERS drug-role, and structured treatment chronology

Agent-specific anti-VEGF co-reporting and drug roles

Agent	Outcome group	N reports	Agent present	Suspect role	Concomitant role	Interacting role
Faricimab	Vasculitis	79	2	2	0	0
Faricimab	Nonvasculitis	571	10	3	7	0
Aflibercept	Vasculitis	79	14	0	14	0
Aflibercept	Nonvasculitis	571	29	1	27	0
Ranibizumab	Vasculitis	79	1	0	0	0
Ranibizumab	Nonvasculitis	571	7	0	7	0
Bevacizumab	Vasculitis	79	2	1	2	0
Bevacizumab	Nonvasculitis	571	5	0	5	0
Brolucizumab	Vasculitis	79	0	0	0	0
Brolucizumab	Nonvasculitis	571	1	1	0	0

Drug-role counts are report-level indicators and may differ from the number of agent-positive reports because a report may contain multiple rows for the same agent and because some rows may have other or missing role codes. Suspect includes primary- or secondary-suspect coding in the derived agent flags used for this summary.

Multiple-agent and faricimab/brolucizumab co-reporting summary

Outcome group	N	Any anti-VEGF	Multiple anti-VEGF agents	Faricimab or brolucizumab
Nonvasculitis	571	50	2	11
Vasculitis	79	19	0	2

Report-level structured chronology summary among anti-VEGF-positive vasculitis reports

Chronology category	N reports	Percent of 19 reports
Not interpretable from structured dates	8	42.1%
Start before/on event only	6	31.6%
Start after event only	3	15.8%
Entries both before/on and after event	2	10.5%

Structured therapy-start and event dates were jointly interpretable for 11 of 19 reports. A start before/on event does not establish active treatment at the time of vasculitis, same-eye exposure, or cumulative injection burden.

Anonymized report-level structured chronology audit

Report	Anti-VEGF agent(s)	FAERS role(s)	Event date available	Therapy start date available	Chronology category
V01	Aflibercept	Concomitant	Yes	No	Not interpretable from structured dates
V02	Aflibercept	Concomitant	Yes	Yes	Start before/on event only
V03	Aflibercept	Concomitant	Yes	No	Not interpretable from structured dates
V04	Aflibercept	Concomitant	Yes	Yes	Start before/on event only
V05	Aflibercept	Concomitant	Yes	Yes	Start before/on event only
V06	Faricimab	Secondary suspect	Yes	Yes	Entries both before/on and after event
V07	Aflibercept	Concomitant	Yes	Yes	Start after event only
V08	Faricimab	Secondary suspect	Yes	Yes	Entries both before/on and after event
V09	Aflibercept	Concomitant	Yes	No	Not interpretable from structured dates
V10	Ranibizumab	Concomitant	Yes	Yes	Start after event only
V11	Aflibercept	Concomitant	Yes	Yes	Start before/on event only
V12	Aflibercept	Concomitant	No	No	Not interpretable from structured dates
V13	Aflibercept	Concomitant	Yes	No	Not interpretable from structured dates
V14	Aflibercept	Concomitant	Yes	Yes	Start after event only
V15	Bevacizumab	Concomitant, Secondary suspect	Yes	Yes	Start before/on event only
V16	Aflibercept	Concomitant	No	No	Not interpretable from structured dates
V17	Bevacizumab	Concomitant	Yes	No	Not interpretable from structured dates
V18	Aflibercept	Concomitant	Yes	Yes	Start before/on event only
V19	Aflibercept	Concomitant	Yes	No	Not interpretable from structured dates

Report labels V01-V19 are arbitrary supplement identifiers and are not FAERS case identifiers. Role codes were summarized from the anti-VEGF rows in the FAERS DRUG table. Multiple structured drug rows can occur within a report.

Supplementary Table S5. Temporal robustness analyses: denominator stress tests and exploratory quarterly Poisson models

Deterministic sensitivity analyses for time-varying denominator error

Scenario	Peg post-denominator multiplier	Far post-denominator multiplier	Peg RR (95% CI)	Far RR (95% CI)	RRR (95% CI)	p value
Far post-denominator multiplier 0.7	1.0	0.7	0.09 (0.05-0.16)	0.17 (0.05-0.52)	0.55 (0.16-1.95)	0.355
Far post-denominator multiplier 0.8	1.0	0.8	0.09 (0.05-0.16)	0.15 (0.05-0.45)	0.63 (0.18-2.23)	0.474
Far post-denominator multiplier 0.9	1.0	0.9	0.09 (0.05-0.16)	0.13 (0.04-0.40)	0.71 (0.20-2.50)	0.594
Far post-denominator multiplier 1.0	1.0	1.0	0.09 (0.05-0.16)	0.12 (0.04-0.36)	0.79 (0.22-2.78)	0.711
Far post-denominator multiplier 1.1	1.0	1.1	0.09 (0.05-0.16)	0.11 (0.03-0.33)	0.87 (0.25-3.06)	0.824
Far post-denominator multiplier 1.2	1.0	1.2	0.09 (0.05-0.16)	0.10 (0.03-0.30)	0.95 (0.27-3.34)	0.931
Far post-denominator multiplier 1.3	1.0	1.3	0.09 (0.05-0.16)	0.09 (0.03-0.28)	1.02 (0.29-3.62)	0.970
Peg post-denominator multiplier 0.8	0.8	1.0	0.12 (0.07-0.20)	0.12 (0.04-0.36)	0.99 (0.28-3.48)	0.981
Peg post-denominator multiplier 0.9	0.9	1.0	0.10 (0.06-0.18)	0.12 (0.04-0.36)	0.88 (0.25-3.09)	0.837
Peg post-denominator multiplier 1.0	1.0	1.0	0.09 (0.05-0.16)	0.12 (0.04-0.36)	0.79 (0.22-2.78)	0.711
Peg post-denominator multiplier 1.1	1.1	1.0	0.08 (0.05-0.15)	0.12 (0.04-0.36)	0.72 (0.20-2.53)	0.604
Peg post-denominator multiplier 1.2	1.2	1.0	0.08 (0.04-0.13)	0.12 (0.04-0.36)	0.66 (0.19-2.32)	0.514
Far fixed all-quarter multiplier 0.7	1.0	0.7	0.09 (0.05-0.16)	0.12 (0.04-0.36)	0.79 (0.22-2.78)	0.711
Far fixed all-quarter multiplier 1.0	1.0	1.0	0.09 (0.05-0.16)	0.12 (0.04-0.36)	0.79 (0.22-2.78)	0.711
Far fixed all-quarter multiplier 1.3	1.0	1.3	0.09 (0.05-0.16)	0.12 (0.04-0.36)	0.79 (0.22-2.78)	0.711

Pre-change denominators were held fixed in the time-varying stress tests. Fixed all-quarter faricimab scaling leaves the within-drug RR and therefore the RRR unchanged because the constant multiplicative factor cancels. Across the prespecified time-varying scenarios, the RRR ranged from approximately 0.55 to 1.02 and every 95% CI included 1.

Quarterly Poisson regression models using log estimated vial exposure as an offset

Model	Interaction term	Rate Ratio (95% CI)	p value	Pearson chi-square/df	Quarter-drug rows	Interpretation
Poisson level-change model	Pegcetacoplan x post-period	0.73 (0.28-1.86)	0.507	1.04	22	Exploratory only. Comparator counts are sparse and only two pre-change quarters are available.
Poisson segmented model	Pegcetacoplan x post-period	2.90 (1.15-7.32)	0.025	0.65	22	Exploratory only. Comparator counts are sparse and only two pre-change quarters are available.
Poisson segmented model	Pegcetacoplan x time after transition	0.71 (0.56-0.89)	0.004	0.65	22	Exploratory only. Comparator counts are sparse and only two pre-change quarters are available.

Q3 2023 was excluded as the transition quarter. The level-change model included drug, calendar quarter, post-recommendation period, and drug-by-post-period interaction. The segmented model additionally included time after the transition quarter and its interaction with drug. Heteroskedasticity-consistent (HC0) sandwich standard errors were used. These models are exploratory because only two pre-change quarters and sparse comparator events were available.

Supplementary Table S6. Event-date completeness and dated-versus-undated report characteristics

Overall availability of quarter-assignable clinical event dates

Cohort	N	Quarter-assignable	Not quarter-assignable
Faricimab	45	14 (31.1%)	31 (68.9%)
Pegcetacoplan	79	58 (73.4%)	21 (26.6%)

Event-date completeness by initial FDA receipt period

Cohort	Receipt period	N	Quarter-assignable
Pegcetacoplan	Pre	6	6 (100.0%)
Pegcetacoplan	Transition	13	11 (84.6%)
Pegcetacoplan	Post	60	41 (68.3%)
Faricimab	Pre	2	1 (50.0%)
Faricimab	Transition	7	4 (57.1%)
Faricimab	Post	36	9 (25.0%)

Characteristics of reports with and without quarter-assignable event dates

Cohort	Date status	N	Coded serious outcome	Strict occlusive-coded	Expanded occlusion-associated	Report-version median (IQR)
Faricimab	Dated	14	12/14 (85.7%)	9/14 (64.3%)	9/14 (64.3%)	2 (1.25-3)
Faricimab	Undated	31	24/31 (77.4%)	15/31 (48.4%)	16/31 (51.6%)	2 (1-3)
Pegcetacoplan	Dated	58	57/58 (98.3%)	28/58 (48.3%)	29/58 (50.0%)	4 (2-5)
Pegcetacoplan	Undated	21	19/21 (90.5%)	14/21 (66.7%)	14/21 (66.7%)	1 (1-3)

Between-status tests: faricimab serious outcome p = 0.698, strict occlusive coding p = 0.356, expanded occlusion-associated coding p = 0.525, report-version number p = 0.503; pegcetacoplan serious outcome p = 0.171, strict occlusive coding p = 0.203, expanded occlusion-associated coding p = 0.212, report-version number p = 0.013. Proportions were compared using two-sided Fisher exact tests and report-version distributions using the Mann-Whitney U test.

Reporter occupation and FAERS report-type distributions by event-date availability

Cohort	Date status	Variable	Category	n	Percent
Faricimab	Dated	Reporter occupation	Physician (MD)	10	71.4%
Faricimab	Dated	Reporter occupation	Other health professional (HP)	3	21.4%
Faricimab	Dated	Reporter occupation	Consumer (CN)	1	7.1%

Faricimab	Undated	Reporter occupation	Physician (MD)	26	83.9%
Faricimab	Undated	Reporter occupation	Other health professional (HP)	2	6.5%
Faricimab	Undated	Reporter occupation	Consumer (CN)	2	6.5%
Faricimab	Undated	Reporter occupation	Missing	1	3.2%
Pegcetacoplan	Dated	Reporter occupation	Physician (MD)	51	87.9%
Pegcetacoplan	Dated	Reporter occupation	Other health professional (HP)	4	6.9%
Pegcetacoplan	Dated	Reporter occupation	Consumer (CN)	3	5.2%
Pegcetacoplan	Undated	Reporter occupation	Physician (MD)	18	85.7%
Pegcetacoplan	Undated	Reporter occupation	Other health professional (HP)	3	14.3%
Faricimab	Dated	Report type	Expedited (EXP)	8	57.1%
Faricimab	Dated	Report type	Periodic (PER)	5	35.7%
Faricimab	Dated	Report type	Direct (DIR)	1	7.1%
Faricimab	Undated	Report type	Expedited (EXP)	20	64.5%
Faricimab	Undated	Report type	Periodic (PER)	11	35.5%
Pegcetacoplan	Dated	Report type	Expedited (EXP)	51	87.9%
Pegcetacoplan	Dated	Report type	Periodic (PER)	5	8.6%
Pegcetacoplan	Dated	Report type	Direct (DIR)	2	3.4%
Pegcetacoplan	Undated	Report type	Expedited (EXP)	16	76.2%
Pegcetacoplan	Undated	Report type	Periodic (PER)	3	14.3%
Pegcetacoplan	Undated	Report type	Direct (DIR)	2	9.5%

Percentages use the number of reports within each cohort/date-status stratum as the denominator. Categories are reported as coded in FAERS.

Supplementary Table S7. Receipt-date sensitivity, reporting lag, and end-of-window analyses

Quarterly retinal vasculitis counts and estimated reporting rates indexed by initial FDA receipt quarter

Quarter	Peg reports	Peg vials	Peg rate/10,000	Far reports	Far vials	Far rate/10,000
Q1 2023	0	9400	0.000	0	182500	0.000
Q2 2023	6	42000	1.429	2	216900	0.092
Q3 2023	13	47000	2.766	7	269600	0.260
Q4 2023	6	62000	0.968	4	301100	0.133
Q1 2024	7	77000	0.909	5	338400	0.148
Q2 2024	9	84000	1.071	3	375300	0.080
Q3 2024	13	88500	1.469	4	403400	0.099
Q4 2024	11	94000	1.170	8	413300	0.194
Q1 2025	1	92000	0.109	1	396700	0.025
Q2 2025	3	95000	0.316	2	404400	0.049
Q3 2025	6	101000	0.594	1	380700	0.026
Q4 2025	4	102000	0.392	8	396700	0.202

Receipt-date pre-change versus post-change analyses

Analysis	Peg pre/post events	Peg RR (95% CI)	Far pre/post events	Far RR (95% CI)	RRR (95% CI)	p value
Receipt-date sensitivity: initial FDA receipt quarter	6/60	0.65 (0.28-1.50)	2/36	2.11 (0.51-8.76)	0.31 (0.06-1.60)	0.161
Receipt-date sensitivity: Q1 2023 excluded	6/60	0.53 (0.23-1.22)	2/36	1.14 (0.28-4.76)	0.46 (0.09-2.41)	0.359

RR indicates post-change versus pre-change reporting rate ratio. RRR is the ratio of the pegcetacoplan RR to the faricimab RR. Q3 2023 was excluded as the transition quarter.

Reporting-lag summary

Cohort	Study-window N	Quarter-assignable N	Quarter-lag N	Quarter lag, median (IQR)	Exact-date N	Lag days, median (IQR)	Negative lag N
Faricimab	45	14	14	1 (1-2.75)	13	72 (52-225)	0
Pegcetacoplan	79	58	58	1 (0-3)	56	79 (27-228)	0

Quarter lag is the difference between clinical event quarter and initial FDA receipt quarter. Exact-day summaries required a quarter-assignable event date and an exact YYYYMMDD clinical event date.

Sensitivity analyses excluding the final 6 months and final 12 months of observation

Analysis	Peg pre/post events	Peg RR (95% CI)	Far pre/post events	Far RR (95% CI)	RRR (95% CI)	p value
Primary through Q2 2025 (final 6 months excluded)	21/30	0.12 (0.07-0.22)	6/6	0.15 (0.05-0.47)	0.82 (0.23-2.88)	0.753

Primary through Q4 2024 (final 12 months excluded)	21/29	0.18 (0.10-0.31)	6/5	0.18 (0.06-0.60)	0.96 (0.26-3.58)	0.955

Supplementary Table S8. Treatment-context sensitivity and report-completeness analyses

Anti-VEGF exposure-definition and clinically restricted sensitivity analyses

Analysis	N	Exposed vasculitis	Exposed nonvasculitis	ROR (95% CI)	Fisher p value
Original any anti-VEGF co-reporting	650	19/79	50/571	3.30 (1.83-5.96)	<0.001
Anti-VEGF coded suspect or concomitant	650	18/79	49/571	3.14 (1.72-5.74)	<0.001
Other anti-VEGF only, exposure excludes faricimab/brolucizumab	650	17/79	40/571	3.64 (1.95-6.80)	<0.001
Drop all reports listing faricimab or brolucizumab; test remaining agents	637	17/77	39/560	3.79 (2.02-7.10)	<0.001
Ocular-adverse-event noncases only	518	19/79	44/439	2.84 (1.56-5.19)	0.001
Retinal/AMD indication-context reports only	407	17/54	34/353	4.31 (2.20-8.46)	<0.001

RORs describe disproportionate representation within Syfovre FAERS reports and do not estimate treated-patient risk.

Documentation characteristics of vasculitis and nonvasculitis reports

Group	N	Drug count, median (IQR)	Reaction count, median	Completeness score, median	Any ocular reaction	Retinal/AMD indication context
Vasculitis	79	4 (2-8)	7	6	100.0%	68.4%
Nonvasculitis	571	1 (1-2)	2	4	76.9%	61.8%

Documentation-adjusted and complete-case demographic logistic models

Model	Term	N	Cases	OR (95% CI)	p value
Anti-VEGF + era + drug count + completeness	Anti-VEGF co-reporting	650	79	1.89 (0.99-3.61)	0.052
Anti-VEGF + era + drug count + completeness	Post-August 2023 reporting era	650	79	0.53 (0.25-1.12)	0.095
Anti-VEGF + era + drug count + completeness	Number of listed drugs	650	79	1.11 (1.05-1.16)	<0.001
Anti-VEGF + era + drug count + completeness	Structured-data completeness score	650	79	1.39 (1.18-1.64)	<0.001
Anti-VEGF + era + age + female complete-case	Anti-VEGF co-reporting	149	27	2.44 (0.78-7.63)	0.126
Anti-VEGF + era + age + female complete-case	Post-August 2023 reporting era	149	27	0.34 (0.14-0.84)	0.020
Anti-VEGF + era + age + female complete-case	Age per 10 years	149	27	0.66 (0.42-1.02)	0.063
Anti-VEGF + era + age + female complete-case	Female sex	149	27	0.90 (0.36-2.28)	0.826

Reaction preferred terms among nonvasculitis Syfovre reports

Reaction preferred term	Number of nonvasculitis reports

Vitreous floaters	101
Visual impairment	91
Choroidal neovascularisation	57
Uveitis	54
Eye inflammation	51
Vitritis	45
Neovascular age-related macular degeneration	45
Vision blurred	44
Death	40
Eye complication associated with device	28
Intra-ocular injection complication	25
Vitreous detachment	24
Iridocyclitis	24
Intraocular pressure increased	23
Blindness	23
Iritis	21
Dry age-related macular degeneration	21
Endophthalmitis	20
Eye pain	19
Visual acuity reduced	18
Ocular hyperaemia	17
Disease progression	16
Retinal haemorrhage	14
Blindness transient	12
Anterior chamber cell	11
Optic ischaemic neuropathy	11

Bacterial endophthalmitis	10
Eye irritation	10
Photophobia	10
Eye haemorrhage	10
Conjunctival haemorrhage	9
Condition aggravated	9
Photopsia	8
Vitreous opacities	8
Ocular discomfort	8
Visual field defect	8
Vitreous haemorrhage	8
Headache	8
Fatigue	8
Eye infection	8
Product dose omission issue	8
Pain	8
Off label use	8
Dry eye	8
Ocular hypertension	7
Drug ineffective	7
Blindness unilateral	7
Intentional dose omission	7
Retinal detachment	6
Lacrimation increased	6

A report may contribute more than one reaction preferred term; counts therefore do not sum to the number of nonvasculitis reports.

General abbreviations: AMD, age-related macular degeneration; anti-VEGF, anti-vascular endothelial growth factor; CHF, Swiss franc; CI, confidence interval; FAERS, US Food and Drug Administration Adverse Event Reporting System; FDA, US Food and Drug Administration; IQR, interquartile range; LR, likelihood ratio; nAMD, neovascular age-related macular degeneration; OR, odds ratio; PT, preferred term; Q1, first quarter; Q2,

second quarter; Q3, third quarter; Q4, fourth quarter; RR, reporting rate ratio; ROR, reporting odds ratio; RRR, ratio of reporting rate ratios; US, United States; USD, US dollar; VEGF, vascular endothelial growth factor; WAC, wholesale acquisition cost.
